# Unconstrained Lagrangian Variational Principles for the Einstein Field Equations

**DOI:** 10.3390/e25020337

**Published:** 2023-02-12

**Authors:** Claudio Cremaschini, Massimo Tessarotto

**Affiliations:** 1 Research Center for Theoretical Physics and Astrophysics, Institute of Physics, Silesian University in Opava, Bezručovo nám.13, CZ-74601 Opava, Czech Republic; 2 Department of Mathematics and Geosciences, University of Trieste, Via Valerio 12, 34127 Trieste, Italy

**Keywords:** Einstein field equations, Lagrangian variational principles, unconstrained variational principles, principle of manifest covariance, 03.50.-z, 03.65-w, 04.20.-q, 04.20.Cv, 04.20.Fy, 04.60-m0101

## Abstract

This paper deals with the problem of establishing a systematic theoretical formulation of variational principles for the continuum gravitational field dynamics of classical General Relativity (GR). In this reference, the existence of multiple Lagrangian functions underlying the Einstein field equations (EFE) but having different physical connotations is pointed out. Given validity of the Principle of Manifest Covariance (PMC), a set of corresponding variational principles can be constructed. These are classified in two categories, respectively, referred to as constrained and unconstrained Lagrangian principles. They differ for the normalization properties required to be satisfied by the variational fields with respect to the analogous conditions holding for the extremal fields. However, it is proved that only the unconstrained framework correctly reproduces EFE as extremal equations. Remarkably, the synchronous variational principle recently discovered belongs to this category. Instead, the constrained class can reproduce the Hilbert–Einstein formulation, although its validity demands unavoidably violation of PMC. In view of the mathematical structure of GR based on tensor representation and its conceptual meaning, it is therefore concluded that the unconstrained variational setting should be regarded as the natural and more fundamental framework for the establishment of the variational theory of EFE and the consequent formulation of consistent Hamiltonian and quantum gravity theories.

## 1. Introduction

The establishment of a theory which yields variational representations of the Einstein field equations (EFE) describing the dynamics of the space-time metric 4-tensor $g_{\mu \nu }$ is a fundamental requisite of field theory and classical General Relativity (GR) in particular. However, due to the consequent intrinsic 4-tensor property acquired by EFE itself, a further possible mathematical requisite arises. This is represented by the 4-tensor “character” of the same variational representation, which means that both the variational Lagrangian, the varied functions as well as the corresponding Euler–Lagrange equations should inherit the same 4-tensor property [1]. Such a requisite, besides being convenient for its simplicity, represents also a consistency property for the logical structure on which physical relativistic field theory (RFT), and in particular classical GR, should be founded [2]. In fact, the same 4-tensor feature warrants by construction the validity of the property of general covariance (a fundamental requisite of RFT) with respect to local point transformations connecting arbitrary different GR-frames $r\equiv \left\{r^{\mu }\right\}$ and $r^{\prime }\equiv \left\{r^{\prime \mu }\right\}$ related by a local diffeomorphism, i.e., of the form given below by Equations (1) and (2). For this reason, the same property and the previous consistency requisite are usually referred to as the Principle of Manifest Covariance (PMC) and PMC requisite [3].

It should be stressed that both requisites are commonly shared by relativistic continuum field theories and relativistic particle dynamics [4]. They should therefore analogously be regarded as physically mandatory in the context of gravitational field theory too. However, despite the fact that both properties are intimately connected with the Einstein theory of General Relativity, rather surprisingly the second requisite is not satisfied by the earliest variational formulation of the Einstein field equations due to Einstein and Hilbert in 1915 [5,6]. As discussed below, the reason can be realized at once by noting that the original Hilbert-Einstein (HE) variational principle misses the coordinate-independence feature. In fact, according to this approach, the volume element appearing in the definition of the variational functional is treated as variational with respect to variations of $g_{\mu \nu }$. As a consequence, despite the 4-scalar property of the variational functional as a whole, the variational Lagrangian is necessarily a 4-scalar density, i.e., it is not a 4-scalar, which therefore implies the violation of the PMC requisite [3].

Besides being a matter of principle, the correct treatment of the variational character of EFE and the related coordinate-independent feature, requires the appropriate formulation of suitable Lagrangian and/or Hamiltonian variational principles for the Einstein field equations, which determine in turn well-defined Lagrangian and Hamiltonian structures [7,8]. Notably, the same structures provide us with the only approach to the formulation of a canonical theory of quantum gravity which is consistent with PMC.

Along this line, the subject of the present paper is to carry out a systematic investigation of the foundations of the theory of variational principles holding for EFE of classical GR. In fact, as shown below, in the literature multiple different Lagrangian functions have been proposed that equivalently generate the same form of extremal gravitational Einstein field dynamical equations. The most relevant ones include, in particular, the original Hilbert–Einstein variational theory, together with the associated Palatini formulation [9,10], the synchronous metric and Ricci principles obtained in the framework of manifestly covariant deDonder–Weyl approach [11,12,13,14], the path-integral synchronous Hamilton variational principle in both unconstrained and constrained forms [15], as well as non-manifestly covariant approaches exemplified by the so-called ADM theory that invoke a slicing of four-dimensional space-time into space and time sub-spaces [16,17,18]. Such a distinction among time and space coordinates, however, might pose conceptual problems on the classical principles of GR. This feature supports the objection raised by Hawking against the ADM theory, who stated that “the split into three spatial dimensions and one time dimension seems to be contrary to the whole spirit of Relativity” [19] (see also Ref. [20] for additional critics on the 3+1 decomposition). In fact, in the spirit of GR, “time” and “space” should be treated on equal footing as independent variables. The distinction among the entries of 4-tensors cannot be longer put on physical basis in GR, in contrast to what happens in flat space-time. As a result, the special role attributed to the “time” (or zero) component with respect to the “space” components does not appear consistently motivated. In addition, the issue can also raise in turn philosofical questions about the quantum meaning of time [21,22].

All these Lagrangian functions are characterized by distinctive physical meanings and mathematical settings of validity, and, apparently, they exhibit independent characters, namely in the sense that their mutual relationships might appear unrelated.

In order to investigate in depth the nature of these variational principles and reach the targets of the research, it is necessary to set up an appropriate theoretical framework for a consistent mathematical treatment. This is obtained by preliminarily introducing the notion of Principle of Manifest Covariance (PMC). In particular, in the framework of a manifestly covariant treatment, PMC states that all dynamical and observable quantities, in particular the Lagrangian functions as well as the continuum Lagrangian coordinates and corresponding operators should be endowed with tensor properties with respect to a suitable group of coordinate transformations. Thus, let us assume for definiteness that the space-time is represented by a Riemannian differential manifold of the type $\left\{Q^{4},\hat{g}\left(r\right)\right\}$, with $Q^{4}$ being the four-dimensional real vector space $R^{4}$ representing the space-time and $\hat{g}\left(r\right)\equiv \left\{{\hat{g}}_{\mu \nu }\left(r\right)\right\}\equiv \left\{{\hat{g}}^{\mu \nu }\left(r\right)\right\}$ being a real and symmetric metric tensor which is parametrized with respect to a coordinate system (or GR-frame) $r\equiv \left\{r^{\mu }\right\}\in Q^{4}$. Then, the same coordinate transformations, denoted as local point transformations (LPT), must preserve the structure of space-time, i.e., they must be realized by local and differentiable bijections of the form

(1) $$r\to r^{\prime }=r^{\prime }\left(r\right),$$

referred to as LPT-group, with inverse

(2) $$r^{\prime }\to r=r\left(r^{\prime }\right),$$

characterized by a non-singular Jacobian matrix $M\equiv \left\{M_{\mu }^{k}\left(r\right)\right\}\equiv \left\{\frac{\partial r^{k}\left(r\right)}{\partial r^{{}^{\prime }\mu }}\right\}$. Thus, $r\equiv \left\{r^{\mu }\right\}$ and $r^{\prime }\equiv \left\{r^{\prime \mu }\right\}$ are arbitrary points belonging to the initial and transformed space-time structures $\left\{Q^{4},\hat{g}\left(r\right)\right\}$ and $\left\{Q^{\prime 4},{\hat{g}}^{\prime }\left(r^{\prime }\right)\right\}$, respectively. The same space-time structure is preserved under the LPT-group, so that actually $\left\{Q^{4},\hat{g}\left(r\right)\right\}\equiv \left\{Q^{\prime 4},{\hat{g}}^{\prime }\left(r^{\prime }\right)\right\}$, while the metric tensors $\hat{g}\left(r\right)$ and ${\hat{g}}^{\prime }\left(r^{\prime }\right)$ transform in each other in accordance with the appropriate 4-tensor transformation laws. More precisely, in tensor form the direct and inverse transformations $\hat{g}\left(r\right)\equiv \left\{{\hat{g}}_{\mu \nu }\left(r\right)\right\}\to {\hat{g}}^{\prime }\left(r^{\prime }\right)\equiv \left\{{\hat{g}}_{\mu \nu }^{\prime }\left(r^{\prime }\right)\right\}$ and ${\hat{g}}^{\prime }\left(r^{\prime }\right)\equiv \left\{{\hat{g}}_{\mu \nu }^{\prime }\left(r^{\prime }\right)\right\}\to \hat{g}\left(r\right)\equiv \left\{{\hat{g}}_{\mu \nu }\left(r\right)\right\}$ read, respectively,

(3) $$\left\{\begin{array}{c} {\hat{g}}_{\alpha \beta }^{\prime }\left(r^{\prime }\right)={\hat{g}}_{\mu \nu }\left(r\left(r^{\prime }\right)\right)\frac{\partial r^{\mu }}{\partial r^{\prime \alpha }}\frac{\partial r^{\nu }}{\partial r^{\prime \beta }}\\ {\hat{g}}_{\mu \nu }\left(r\right)={\hat{g}}_{\alpha \beta }^{\prime }\left(r^{\prime }\left(r\right)\right)\frac{\partial r^{\prime \alpha }}{\partial r^{\mu }}\frac{\partial r^{\prime \beta }}{\partial r^{\nu }} \end{array}\right.,$$

while the metric tensor fields $\hat{g}\left(r\right)$ and ${\hat{g}}^{\prime }\left(r^{\prime }\right)$ are required to satisfy the orthogonality conditions

(4) $$\begin{array}{ccc} {\hat{g}}_{\mu \nu }\left(r\right){\hat{g}}^{\mu \eta }\left(r\right) & = & \delta_{\nu }^{\eta }, \end{array}$$

(5) $$\begin{array}{ccc} {\hat{g}}_{\mu \nu }^{\prime }\left(r^{\prime }\right){\hat{g}}^{\prime \mu \eta }\left(r^{\prime }\right) & = & \delta_{\nu }^{\eta }. \end{array}$$

Finally, the Riemann distance in the two space-times $\left\{Q^{4},\hat{g}\left(r\right)\right\}$ and $\left\{Q^{\prime 4},{\hat{g}}^{\prime }\left(r^{\prime }\right)\right\}$ is the same, namely it is realized by means of a 4-scalar, so that $ds^{2}={\hat{g}}_{\mu \nu }\left(r\right)dr^{\mu }dr^{\nu }={\hat{g}}_{\mu \nu }^{\prime }\left(r^{\prime }\right)dr^{\prime \mu }dr^{\prime \nu }$, while any other 4-tensor, including the Ricci and Riemann tensors, transforms in accordance with the well-known covariance 4-tensor transformation laws [10].

Given these premises, one of the main goals of the current research is to prove that, precisely under validity of PMC, a comprehensive classification of main literature variational principles can be given. This is realized by pointing out the existence of two categories to which the latter principles belong, respectively, referred to as constrained and unconstrained Lagrangian principles. A general mathematical procedure for the determination of each variational approach is formulated. This in turn permits to unveil the difference existing between the two sets of corresponding variational principles. In fact, it is shown that this lies in the physical connotation that characterizes the generalized Lagrangian coordinates, with particular emphasis on the normalization and/or orthogonality properties required to be satisfied by the variational fields with respect to the analogous conditions holding for the extremal fields. The treatment is developed initially for the case of vacuum Einstein equations, namely without external source fields, but with inclusion of the cosmological constant term. The discussion about the extension of the formalism to the treatment of external sources (i.e., the non-vacuum case) is then completed in a subsequent separate section.

As a notable outcome, it is proved that only the unconstrained variational approach provides a correct framework of general validity able to reproduce EFE as extremal equations. Among the possible realizations, this includes the synchronous Lagrangian variational principle disclosed in Ref. [23]. Instead, it is shown that the constrained class can reproduce the Hilbert–Einstein formulation, which therefore realizes effectively a constrained variational principle. The validity of such a derivation however demands unavoidably violation of PMC. This provides a novel point of view on the physical origin of the HE variational principle and its relationship with manifest covariance principle [3]. On the other hand, this conclusion precludes the applicability of the constrained setting with respect to the unconstrained one. This should be therefore regarded as the unique and preferred variational framework for its consistency with the mathematical structure of GR rooted on PMC and the tensor representation of observables and dynamical equations. Hence, the theory of unconstrained variational principles promotes itself as the only viable way for the establishment of Lagrangian approaches to classical GR that can permit also the establishment of a related Hamiltonian formulation and, ultimately, to reach a consistent theory of quantum gravity [24].

The present research is aimed at providing a novel theoretical framework for the development of variational principles at the basis of classical GR and the understanding of their physical significance. The subject in fact still represents a fertile field of research in mathematical physics, General Relativity, field theory and Quantum Gravity, with potential applications that might involve also the search of alternative gravitational theories beyond classical GR. The motivations at the basis of the proposed research can be identified with the following issues:

(1) The investigation of the role and conditions of applicability of PMC at the level of variational principles for GR equations. In fact, the same principle is a pillar of the foundational scheme of GR theory and the related tensorial representation of EFE. Therefore, one should recover validity of PMC also at the variational level, namely characterizing the definitions of variational principles, action functionals and Lagrangian fields associated with the GR equations. The aim is to understand how PMC can be realized for consistency in the variational domain and how this requirement places itself with respect to the properties of alternative GR variational principles available in the literature.

(2) The physical interpretation of variational fields yielding GR equations in the framework of PMC, in connection with the geometrical interpretation of metric tensor. In fact, GR distinguishes itself from other continuum field theories for the fact that it determines the gravitational field, its dynamics and simultaneously also the space-time geometry. Namely, this refers to the background space-time on which the same field dynamics is realized and in which interaction with other fields takes place. Because of this physical and geometrical connotation, there remains to be ascertained how PMC can be effectively realized at variational level when the notion of background (i.e., extremal) metric tensor is required for its definition.

(3) To provide a classification of the variational approaches for EFE available in the literature and point out their mutual relationship, namely their common aspects as well as intrinsic differences. The task is met by means of the introduction of the concept of constrained and unconstrained variational principles. In particular, one of the main outcome is to disclose the existence of a novel class of unconstrained Lagrangian variational principles underlying the GR equations. The relevance of this kind of principles with respect to previous literature constrained principles is that they can be used to construct corresponding unconstrained Hamiltonian formulations for EFE. In view of this application, the advantage of the unconstrained approach lies in the fact that the unconstrained Hamiltonian structure provides a viable setting for the construction of a quantum gravity theory.

(4) To establish a connection with classical mechanics and continuum field theory for what concerns the identification of Lagrangian and Hamiltonian variational principles and their formal representations.

## 2. Variational Approaches to EFE in the Literature

During the past decades, several alternative variational approaches were proposed in this regard. Historically, the original formulation of the problem was reached in terms of the Hilbert–Einstein (HE) variational theory [5], which is based on the action functional

(6) $$S_{HE}\left(g\left(r\right)\right)\equiv \int_{Q^{4}}d\Omega L_{HE}\left(g\right),$$

where $d\Omega \equiv d^{4}r\delta \sqrt{-\left|g(r)\right|}$ is the invariant 4-volume element of the Riemann space-time $\left\{Q^{4},g\left(r\right)\right\}$, with $d^{4}r\equiv \prod_{i=0,3}dr^{i}$ being the canonical measure of $Q^{4}$, and $\left|g(r)\right|$ denoting here the determinant of g(r). Furthermore, $L_{HE}\left(g\right)$ denotes the HE Lagrangian 4-scalar function defined as

(7) $$L_{HE}\left(g\right)=V_{HE}\left(g\right)+V_{F},$$

where

(8) $$V_{HE}\left(g\right)=-\frac{c^{3}}{16\pi G}\left(g^{\mu \nu }R_{\mu \nu }\left(g\right)-2\Lambda \right),$$

with Λ>0, *G* and *c* being, respectively, the cosmological constant, the Newton constant of gravitation and the speed of light in vacuum. Instead, $V_{F}\equiv V_{F}\left(g,r\right)$ is the non-vacuum contribution due to possible external fields to be prescribed in terms of the field Lagrangian $L_{F}$ as $V_{F}=\frac{1}{c}L_{F}$. Hence, the quantity $\sqrt{-\left|g\right|}L_{HE}\left(g\right)$ identifies the corresponding variational Lagrangian density. According to the HE theory, the action $S_{HE}\left(g\left(r\right)\right)$ is considered dependent only on the variational field $g\left(r\right)\equiv \left\{g_{\mu \nu }\right\}$, whose independent 4-tensor components represent the generalized Lagrangian coordinates. Each g(r) belongs to a suitably constrained functional setting ${\left\{g(r)\right\}}_{C}$[3]. In fact, any 4-tensor $g\left(r\right)\in {\left\{g(r)\right\}}_{C}$ also realizes a metric tensor, so that its countervariant and covariant components respectively raise and lower tensor indices and thus necessarily must satisfy the orthogonality condition $g_{\mu \nu }g^{\mu k}=\delta_{\nu }^{k}$, implying in turn the “normalization” condition $g_{\mu \nu }\left(r\right)g^{\mu \nu }\left(r\right)=4$. For the same reason, in the functional setting ${\left\{g(r)\right\}}_{C}$, the tensor g(r) necessarily must also determine the Christoffel symbols Γ(g(r)) and the Ricci tensor $R_{\mu \nu }\left(g\right)$, so that g(r) satisfies the metric compatibility condition with vanishing covariant derivatives.

The asynchronous HE approach is characterized by a number of critical aspects which rise potential mathematical and conceptual divergences both with standard variational theory of continuum classical fields and the postulates of GR. These issues pertain primarily:

(1) The validity of the principle of manifest covariance, since the HE variational Lagrangian density is not a 4-scalar because of the presence of the determinant $\left|g(r)\right|$.

(2) The non-standard character of $L_{HE}\left(g\right)$ which depends on second-order partial derivatives of Lagrangian coordinate $g_{\mu \nu }\left(r\right)$ through the variations of the non-linear Ricci tensor contributions. This means that the HE variational principle is not cast in so-called first-order Lagrangian formalism. As a consequence, an appropriate treatment of differential fixed-point boundary terms generated in this way is required. Proposals of this kind can be found in Refs. [9,10,25], while Ref. [3] provides a novel conceptually new point of view for an alternative overcome of the problem that restores the customary first-order formulation of least-action principles.

(3) The related missing canonical structure of the HE Lagrangian that is not expressed as a customary sum of “kinetic” and “potential” terms. In contrast, the Ricci scalar can be viewed as a sort of coupling term between the metric tensor and the Ricci tensor, a feature which by itself appears peculiar in the framework of classical variational theory for continuum fields.

(4) The violation of the fundamental gauge invariance properties to be satisfied by the HE Lagrangian function [3,23].

In detail, concerning the variational calculus, the HE variational principle is expressed by the requirement that for arbitrary variations δg(r) it must be

(9) $${\left.\delta S_{HE}\left(g\left(r\right)\right)\right|}_{g=\hat{g}\left(r\right)}={\left.\frac{d}{d\theta }S_{HE}\left(\hat{g}\left(r\right)+\theta \delta g\left(r\right)\right)\right|}_{\theta =0}=0,$$

with the symbol δ denoting the Frechet derivative and $\hat{g}\left(r\right)$ being the extremal classical metric tensor, to be identified “a posteriori” with the solution of EFE. A characteristic feature of the HE variational theory is that dΩ yields non-vanishing variational contributions to $\delta S_{HE}\left(g\left(r\right)\right)$, since $\delta d\Omega =d^{4}r\delta \sqrt{-\left|g\right|}$, where $\delta \sqrt{-\left|g\right|}=\frac{1}{2}\sqrt{-\left|g\right|}g^{\mu \nu }\delta g_{\mu \nu }$. This means that the variation of the functional $S_{HE}\left(g\left(r\right)\right)$ does not preserve the space-time volume element. Because of formal analogies of this property with the analogous occurrence arising in non-relativistic classical mechanics and adopting a similar nomenclature, the HE variational principle is referred to as asynchronous [23]. One obtains that

(10) $${\left.\delta S_{HE}\left(g\left(r\right)\right)\right|}_{g=\hat{g}\left(r\right)}={\left.\delta S_{HE}\left(g\right)\right|}_{\mathrm{expl}}+{\left.\delta S_{HE}\left(g\right)\right|}_{\mathrm{impl}},$$

where the implicit contribution is

(11) $${\left.\delta S_{HE}\left(g\right)\right|}_{\mathrm{impl}}=\int_{Q^{4}}d\Omega \left[-\frac{c^{3}}{16\pi G}{\hat{g}}^{\alpha \beta }\frac{\delta R_{\alpha \beta }}{\delta g^{\mu \nu }}\right]\delta g^{\mu \nu },$$

while the explicit contributions can be written as

(12) $${\left.\delta S_{HE}\left(g\right)\right|}_{\mathrm{expl}}=\int_{Q^{4}}d^{4}r\left[A_{\mu \nu }+B_{\mu \nu }+C_{\mu \nu }\right]\delta g^{\mu \nu },$$

where $A_{\mu \nu }$, $B_{\mu \nu }$ and $C_{\mu \nu }$ are tensor densities defined as

(13) $$\begin{array}{ccc} A_{\mu \nu } & \equiv & L_{HE}\frac{\delta \sqrt{-\left|g\right|}}{\delta g^{\mu \nu }}, \end{array}$$

(14) $$\begin{array}{ccc} B_{\mu \nu } & \equiv & -\frac{c^{3}}{16\pi G}\sqrt{-\left|g\right|}R_{\alpha \beta }\frac{\delta g^{\alpha \beta }}{\delta g^{\mu \nu }}, \end{array}$$

(15) $$\begin{array}{ccc} C_{\mu \nu } & \equiv & \frac{1}{c}\sqrt{-\left|g\right|}\frac{\delta L_{F}}{\delta g^{\mu \nu }}. \end{array}$$

As shown in Ref. [3], in order to recover the correct form of EFE, the constraint condition ${\left.\delta S_{HE}\left(g\right)\right|}_{\mathrm{impl}}=0$ must hold for arbitrary variations $\delta g^{\mu \nu }\left(r\right)$, so that the explicit contributions are sufficient to yield the correct Einstein equations

(16) $${\hat{R}}_{\mu \nu }-\frac{1}{2}\hat{R}{\hat{g}}_{\mu \nu }+\Lambda {\hat{g}}_{\mu \nu }=\kappa {\hat{T}}_{\mu \nu },$$

where ${\hat{R}}_{\mu \nu }=$$R_{\mu \nu }\left(\hat{g}\left(r\right)\right)$ and $\hat{R}={\hat{g}}^{\mu \nu }\left(r\right){\hat{R}}_{\mu \nu }\equiv R\left(\hat{g}\left(r\right)\right)$ denote, respectively, the background Ricci 4-tensor and Ricci 4-scalar, ${\hat{T}}_{\mu \nu }=$$T_{\mu \nu }\left(\hat{g}\left(r\right)\right)$ is the background stress-energy tensor associated with the external source fields described by the external-field Lagrangian density $L_{F}\left(g\right)$, and κ denotes the universal constant $\kappa =8\pi G/c^{4}$.

It must be stressed that multiple equivalent representations of the HE variational theory were proposed in the literature starting from the initial work by Einstein, which preserve the standard formulation of classical GR equations. These include in particular:

(1) Approaches such as those reported in Refs. [9,10,25] already mentioned above, which differ for the way of treating fixed-points and boundary terms arising from variational calculus of the HE Lagrangian.

(2) Geometrical approaches referred to as tetrad formalism of differential geometry, in which the metric tensor is represented in terms of so-called vielbeins [26,27].

(3) Non-manifestly covariant approaches, such as the Dirac approach, the ADM theory and generally any 3+1 formulation based on preliminary decomposition of space-time into the product of one time-like dimension and a three-space slice [16,17,18].

In addition, the quest for restoring a first-order formalism of the HE theory and warranting at the same time a proper treatment of the Ricci-tensor variational contributions led to the discovery of the so-called Palatini formulation of variational GR [9], in which the differential connections (equivalently taken as the component fields of the covariant derivative [25]) are treated as independent variational fields besides the metric tensor. The resulting extremal equations are then identified with EFE plus the metric compatibility condition prescribing the Christoffel symbols. However, this approach is intrinsically non-manifestly covariant, since the connections do not possess by definition a tensorial character. This feature can be viewed as another way of expressing the peculiar role taken by the Ricci tensor in the HE variational theory, which depends on second-order partial derivatives of the metric tensor and therefore ultimately does not permit to satisfy the first-order formalism of standard variational theories.

More recently, a novel approach to the issue, consistent with the deDonder–Weyl manifestly covariant variational theory for continuum fields [11,12], has been proposed. This is provided by the synchronous variational principle for the gravitational field outlined in Refs. [13,23]. The latter has the advantage of overcoming the problems of the HE theory related to the violation of both manifest covariance and the first-order formalism. In fact, the synchronous variational approach is characterized by a 4-scalar Lagrangian function expressed in terms of superabundant variables $g_{\mu \nu }$ and ${\hat{g}}_{\mu \nu }$. In this setting, the variational tensor $g\equiv \left\{g_{\mu \nu }\right\}$ is distinguished from a non-variational background metric tensor $\hat{g}\equiv \left\{{\hat{g}}_{\mu \nu }\right\}$, which defines the covariance properties of the theory and is ultimately assumed to be determined “a posteriori” by the extremal EFE. Hence, $\hat{g}$ expresses the geometric character of the metric tensor, namely it satisfies the orthogonality condition ${\hat{g}}_{\mu \nu }{\hat{g}}^{\mu k}=\delta_{\nu }^{k}$, so that it raises/lowers tensor indices, as well the metric compatibility condition ${\hat{\nabla }}_{\alpha }{\hat{g}}_{\mu \nu }=0$, so that it defines the standard Christoffel connections and curvature tensors of space-time. On the contrary, in this framework the variational tensor *g* is such that $g_{\mu \nu }g^{\mu k}\neq \delta_{\nu }^{k}$. The distinction between *g* and $\hat{g}$ holds only at the variational level, since in the (extremal) EFE the identity $g=\hat{g}$ is restored. In the synchronous setting, hatted quantities depend on the background metric tensor $\hat{g}$ and do not contribute to the variational calculus. Thus, denoting in particular the synchronous volume element as $d\hat{\Omega }=d^{4}r\sqrt{-\left|\hat{g}\right|}$, its variation vanishes by construction so that $\delta d\hat{\Omega }=0$. This volume-preserving property under the action of the operator δ justifies the name given to this approach as the synchronous variational principle, in contrast to the asynchronous theory. For completeness, it must be noted that the synchronous setting exhibits similarities with other relevant literature approaches known as non-metric volume forms, or modified measures, defined for example in Refs. [28,29], or the so-called non-Riemannian space-time volume elements [30]. These works proposed variational models for the GR equations in which the volume elements of integration in the action principles are metric independent and are rather determined dynamically through additional degrees of freedom, such as the inclusion of additional four scalar fields. Therefore, both synchronous and non-metric approaches do not treat the volume element of integration as a variational quantity depending on a variational metric tensor. This feature certainly represents a breakthrough in the variational approach to EFE with respect to other literature models. However, the synchronous setting remains distiunguished because it does not rely on inclusion, nor does it predict the onset of additional fields, but only the use of superabundant field variables which nevertheless coincide with the unique observable space-time metric tensor in the extremal Einstein equations.

The synchronous Lagrangian action functional is defined as

(17) $$S_{s}\left(g\left(r\right),\hat{g}\left(r\right)\right)=\int_{Q^{4}}d\hat{\Omega }L_{s}\left(g,\hat{g}\right),$$

where $S_{s}\left(g\left(r\right),\hat{g}\left(r\right)\right)$ is considered a functional dependent only on the variational tensor (not a metric tensor) $g\left(r\right)\equiv \left\{g_{\mu \nu }\right\}$.Here, $L_{s}\left(g,\hat{g}\right)\equiv L_{s}\left(g\left(r\right),\hat{g}\left(r\right)\right)$ is the variational Lagrangian and, in contrast to the asynchronous action functional (6), the volume element takes the form $d\hat{\Omega }$. The variational Lagrangian is written as

(18) $$L_{s}\left(g,\hat{g}\right)\equiv h\left(g,\hat{g}\right)L\left(g,\hat{g}\right),$$

where the 4-scalar

(19) $$h\left(g,\hat{g}\right)=2-\frac{1}{4}g^{\eta \beta }\left(r\right)g^{\mu \nu }\left(r\right){\hat{g}}_{\eta \mu }\left(r\right){\hat{g}}_{\beta \nu }\left(r\right)$$

identifies the variational weight-factor defined so that $h(\hat{g},\hat{g})=1$. Instead, the 4-scalar Lagrangian $L(g,\hat{g})$ takes the form

(20) $$L\left(g,\hat{g}\right)=V_{G}\left(g,\hat{g}\right)+V_{F}\left(g,\hat{g}\right),$$

where now

(21) $$\begin{array}{ccc} V_{G}\left(g,\hat{g}\right) & = & -\frac{c^{3}}{16\pi G}\left(g^{\mu \nu }{\hat{R}}_{\mu \nu }-2\Lambda \right), \end{array}$$

(22) $$\begin{array}{ccc} {\hat{R}}_{\mu \nu } & \equiv & R_{\mu \nu }\left(\hat{g}\right), \end{array}$$

and $V_{F}\left(g,\hat{g}\right)=\frac{1}{c}L_{F}\left(g,\hat{g}\right)$. Then, the synchronous Lagrangian action principle follows by prescribing

(23) $${\left.\delta S_{s}\left(g\left(r\right),\hat{g}\left(r\right)\right)\right|}_{g=\hat{g}}=0,$$

for arbitrary variations δg(r), while noting that $\delta \hat{g}\left(r\right)\equiv 0$. Here, the symbol δ denotes the variation operator, i.e., the Frechet derivative

(24) $${\left.\delta S_{s}\left(g\left(r\right),\hat{g}\left(r\right)\right)\right|}_{g=\hat{g}}\equiv {\left.\frac{d}{d\theta }S_{L}\left(\hat{g}\left(r\right)+\theta \delta g\left(r\right),\hat{g}\left(r\right)\right)\right|}_{\theta =0}.$$

By noting that $\delta h\left(g,\hat{g}\right)=-\frac{1}{2}{\hat{g}}^{\mu \nu }\left(r\right)\delta g_{\mu \nu }$, the evaluation of ${\left.\delta S_{s}\left(g\left(r\right),\hat{g}\left(r\right)\right)\right|}_{g=\hat{g}\left(r\right)}$ is straightforward. In fact, in the synchronous setting, only explicit dependences on *g* give a contribution, while the implicit ones carried by the Ricci tensor are now excluded. Hence, from Equation (23) one recovers EFE in the correct form (16).

Finally, in view of the following developments, it is worth recalling another Lagrangian formulation to EFE recently proved to hold and reported in Ref. [14]. This is based on the implementation of the principle of manifest covariance, whereby the independent variational field in the Lagrangian variational principle (i.e., the Lagrangian generalized coordinate field) becomes identified with the Ricci tensor $R_{\mu \nu }$ in place of the (metric) tensor $g_{\mu \nu }$. More precisely, in such a framework the realization of a consistent variational principle requires the identification of the functional as

(25) $$S_{R}\left(\hat{g}\left(r\right),R\left(r\right)\right)\equiv \int_{Q^{4}}d\hat{\Omega }L_{R}\left(\hat{g},R\right),$$

to be denoted as Ricci-functional. The latter is considered to depend only on the variational tensor field $R\left(r\right)\equiv \left\{R_{\mu \nu }\left(r\right)\right\}$, with $R\left(r\right)$ belonging to a suitable synchronous variational setting, while here the 4-tensor field $\hat{g}\left(r\right)$ identifies the prescribed metric field tensor solution of EFE, to be considered effectively as a metric tensor. The 4-scalar variational Ricci Lagrangian function $L_{R}\left(\hat{g},R\right)$ is taken of the form

(26) $$L_{R}\left(\hat{g},R\right)\equiv -\frac{c^{3}}{16\pi G}\left(\frac{1}{2\Lambda }\rho +R-\frac{1}{4\Lambda }R^{2}\right)+\frac{1}{2c}\frac{1}{\Lambda }R^{\mu \nu }{\hat{T}}_{\mu \nu },$$

with $\rho \equiv R^{\mu \nu }R_{\mu \nu }$, and $R\equiv R^{\mu \nu }{\hat{g}}_{\mu \nu }$ denoting the corresponding variational Ricci 4-scalar. We notice that the dimensional units are set so to make the Lagrangian $L_{R}\left(\hat{g},R\right)$ homogeneous with $L_{HE}$ and $L_{s}$, warranting that $L_{R}\left(\hat{g},R\right)$ is an action. Accordingly, the stress-energy tensor ${\hat{T}}_{\mu \nu }$ has the same dimension of the external-field Lagrangian $L_{F}$ introduced above. However, different from previous realizations of Lagrangian functions, now the Lagrangian $L_{R}\left(\hat{g},R\right)$ is a polynomial function that contains a linear and a quadratic contribution in the Ricci 4-scalar *R*, a quadratic term in the Ricci tensor $R^{\mu \nu }$ which enters through the curvature 4-scalar $\rho \equiv R^{\mu \nu }R_{\mu \nu }$ and a linear term in the Ricci tensor $R^{\mu \nu }$ that carries the coupling with external sources. The Ricci Lagrangian variational principle associated with the action integral $S_{R}\left(\hat{g}\left(r\right),R\left(r\right)\right)$ can then be obtained by requiring that for arbitrary variations $\delta R\left(r\right)\equiv \delta R^{\mu \nu }\left(r\right)$, it must be

(27) $${\left.\delta S_{R}\left(\hat{g}\left(r\right),R\left(r\right)\right)\right|}_{R=\hat{R}\left(r\right)}={\left.\frac{d}{d\theta }S_{R}\left(\hat{R}\left(r\right)+\theta \delta R\left(r\right)\right)\right|}_{\theta =0}=0,$$

with the symbol δ denoting again the Frechet derivative and the variation being performed with respect to the independent tensor field $R^{\mu \nu }\left(r\right)$. It is then immediate to verify that the explicit algebraic calculation of the previous equation yields Equation (16) correctly.

This completes the short review of the most relevant literature approaches proposed in the past for the establishment of a variational formulation of EFE in classical General Relativity. Their presentation and comment of main physical and mathematical properties are useful for the following establishment of a general classification of Lagrangian variational principles, to be carried out in terms of suitably defined constrained and unconstrained principles, and to point out their mutual relationship.

## 3. General Formalism for Lagrangian Principles

In this section, we propose a theoretical method that permits one to obtain a Lagrangian variational formulation of EFE which is necessarily consistent with PMC. This means that the definition of the Lagrangian function and variational Lagrangian fields are such to be always expressed in terms of 4-tensor or 4-scalar fields. The technique developed here is instrumental for the following treatment as it provides the appropriate framework for the distinction between constrained and unconstrained variational principles. In addition, the same approach is general enough to be comprehensive of the variational formulations developed in the previous literature and allows for a novel physical interpretation of their relevance and mutual meaning.

The starting point is represented by the classical form of the vacuum tensor EFE with non-vanishing cosmological constant Λ, which follow from Equation (16) setting ${\hat{T}}_{\mu \nu }=0$. As said above, in the first instance the treatment is restricted to the vacuum case in order to single out the salient features of the theory. The extension to the inclusion of source fields through the stress-energy tensor ${\hat{T}}_{\mu \nu }$ appearing on the rhs of EFE will be discussed below in a separate section in order to also provide a comprehensive comparison on how the issue is handled among the different constrained and unconstrained variational approaches. However, for later convenience, we define the covariant Einstein tensor $G_{\mu \nu }$ as

(28) $$G_{\mu \nu }\equiv {\hat{R}}_{\mu \nu }-\frac{1}{2}\hat{R}{\hat{g}}_{\mu \nu }+\Lambda {\hat{g}}_{\mu \nu },$$

so that EFE can be written equivalently in compact form as

(29) $$G_{\mu \nu }=0.$$

Then, invoking PMC, we construct a 4-scalar *Z* out of the tensor $G_{\mu \nu }$ by index saturation with a generic second-order controvariant symmetric tensor $Z^{\mu \nu }$, namely

(30) $$Z\equiv Z^{\mu \nu }G_{\mu \nu }.$$

The representation of the tensor $Z^{\mu \nu }$ selects the kind of variational principle to be dealt with and, without restrictions, it can be identified with any physically meaningful second-rank tensor of GR. This issue will be exemplified in detail below.

In order to obtain the sought Lagrangian function, we then promote the tensor $Z^{\mu \nu }$ to be the variational tensor. As a consequence, in case the controvariant tensor $Z^{\mu \nu }$ coincides with one of the covariant tensors appearing in $G_{\mu \nu }$, namely one of the tensors of the set ${\hat{z}}_{\mu \nu }=$(${\hat{R}}_{\mu \nu },{\hat{g}}_{\mu \nu }$), the latter must be assumed as well to be variational. In such a case, we denote explicitly the dependence

(31) $$G_{\mu \nu }=G_{\mu \nu }\left(Z_{\mu \nu },\left\{{\hat{z}}_{\mu \nu }-{\hat{Z}}_{\mu \nu }\right\}\right),$$

where $\left\{{\hat{z}}_{\mu \nu }-{\hat{Z}}_{\mu \nu }\right\}$ stands for the set ${\hat{z}}_{\mu \nu }$ with the exclusion of the only tensor ${\hat{Z}}_{\mu \nu }$. This warrants that equal tensors entering the scalar product in Equation (30) are always variational in both covariant and controvariant components. A fundamental element at this stage concerns the definition of the variational functional class $\left\{Z\right\}$ to which $Z^{\mu \nu }$ belongs, to be necessarily assigned for consistency with PMC. On general grounds, it expresses the physical properties satisfied by the variational tensor $Z^{\mu \nu }$ and/or its extremal counterpart ${\hat{Z}}^{\mu \nu }$, as well as possibly by corresponding differential tensors, to be denoted symbolically as $\partial Z^{\mu \nu }$. Remarkably, the mathematical representation of these tensorial properties can equally identify suitable functional constraints for the variational fields and, in turn, also for the same variational principle. In compact notation, the functional class $\left\{Z\right\}$ can then be written as

(32) $$\left\{Z\right\}\equiv \left\{f_{i}\left(Z^{\mu \nu },{\hat{Z}}^{\mu \nu },\partial Z^{\mu \nu }\right)=0,i=1,k\right\}.$$

However, we notice that it is not sufficient to trivially identify the Lagrangian function with the 4-scalar *Z*. In fact, in order to warrant the procedure to work in full generality and allow the formalism to reproduce the different literature formulations, the Lagrangian function $L_{Z}$ must be assumed of the form

(33) $$L_{Z}=L_{Z}\left(Z,\alpha_{i}\right).$$

Here, $\alpha_{i}$, with i=1,3, identifies a suitable set of non-variational constant coefficients. Their value must be set “a posteriori” to warrant the identity of extremal field equations with EFE for each variational principle realized by the Lagrangian functions $L_{Z}$. As shown below, the solution for $\alpha_{i}$ depends uniquely on the actual identification of the tensor $Z^{\mu \nu }$ and the fundamental definition of the variational functional class $\left\{Z\right\}$.

Then, we introduce the action functional

(34) $$S_{Z}\equiv \int_{Q^{4}}d\Omega L_{Z}\left(Z,\alpha_{i}\right),$$

where $d\Omega \equiv d^{4}r\delta \sqrt{-\left|\hat{g}\right|}$ is the invariant 4-volume element of the Riemann space-time $\left\{Q^{4},g\left(r\right)\right\}$ and $d^{4}r\equiv \prod_{i=0,3}dr^{i}$ its canonical measure. The corresponding variational principle is then obtained by requiring that for arbitrary variations $\delta Z^{\mu \nu }$ belonging to $\left\{Z\right\}$ it must be

(35) $$\delta S_{Z}={\left.\frac{d}{d\theta }S_{Z}\left(L_{Z}\left(Z^{\mu \nu }+\theta Z^{\mu \nu },\alpha_{i}\right)\right)\right|}_{\theta =0}=0,$$

with the symbol δ denoting the Frechet derivative. It must be stressed that this procedure warrants the validity of PMC, which is found to be always satisfied identically according to the previous definitions.

## 4. Unconstrained Ricci Lagrangian Principle

We consider a first application of the method outlined above. This concerns the construction of so-called Ricci Lagrangian, namely the variational principle in which the variational field is identified with the Ricci tensor. For consistency with PMC, the latter field is regarded as an independent tensor, thus ignoring its functional dependence in terms of Christoffel connections and partial derivatives of the metric tensor. Following the prescriptions indicated in the previous section, we then start by assuming the identification

(36) $$Z^{\mu \nu }=R^{\mu \nu },$$

where $R^{\mu \nu }$ denotes the variational Ricci tensor, with the corresponding extremal tensor denoted as ${\hat{R}}^{\mu \nu }$. The evaluation of the 4-scalar *Z* according to Equation (30) then requires considering the covariant Ricci tensor $R_{\mu \nu }$ in $G_{\mu \nu }$ to be variational as well, so that $Z_{\mu \nu }=R_{\mu \nu }$, while $\left\{{\hat{z}}_{\mu \nu }-{\hat{Z}}_{\mu \nu }\right\}=\left\{{\hat{g}}_{\mu \nu }\right\}$. The Ricci scalar becomes accordingly $R=R^{\alpha \beta }{\hat{g}}_{\alpha \beta }$. Hence, we have formally that

(37) $$G_{\mu \nu }=G_{\mu \nu }\left(R_{\mu \nu },{\hat{g}}_{\mu \nu }\right),$$

and correspondingly

(38) $$Z=R^{\mu \nu }G_{\mu \nu }\left(R_{\mu \nu },{\hat{g}}_{\mu \nu }\right).$$

Explicit calculation then gives

(39) $$Z=R^{\mu \nu }R_{\mu \nu }-\frac{1}{2}\left(R^{\alpha \beta }{\hat{g}}_{\alpha \beta }\right)R^{\mu \nu }{\hat{g}}_{\mu \nu }+\Lambda R^{\mu \nu }{\hat{g}}_{\mu \nu },$$

namely

(40) $$Z=\rho -\frac{1}{2}R^{2}+\Lambda R,$$

where we have introduced the compact notation $\rho \equiv R^{\mu \nu }R_{\mu \nu }$ and again $R=R^{\alpha \beta }{\hat{g}}_{\alpha \beta }$.

The Ricci Lagrangian function is identified with

(41) $$L_{Z}\left(Z,\alpha_{i}\right)\to L_{R}=L_{R}\left(R^{\mu \nu },\alpha_{i}\right),$$

where

(42) $$L_{R}\left(R^{\mu \nu },\alpha_{i}\right)=\alpha_{1}\rho -\alpha_{2}\frac{1}{2}R^{2}+\alpha_{3}\Lambda R.$$

Adopting analogous notation, the action integral becomes

(43) $$S_{R}\equiv \int_{Q^{4}}d\Omega L_{R}\left(R^{\mu \nu },\alpha_{i}\right),$$

with $L_{R}\left(R^{\mu \nu },\alpha_{i}\right)$ being given by Equation (42). The functional class of variations $\left\{Z\right\}\to \left\{R\right\}$ is defined as

(44) $$\left\{R\right\}\equiv \left\{\begin{array}{c} Z^{\mu \nu }\equiv R^{\mu \nu }\\ R_{\mu \nu }=R^{\alpha \beta }{\hat{g}}_{\mu \alpha }{\hat{g}}_{\nu \beta }\\ {\left.R_{\mu \nu }\right|}_{{\hat{g}}_{\mu \nu }}\equiv {\hat{R}}_{\mu \nu } \end{array}\right\}.$$

We notice the remarkable feature that in the class $\left\{R\right\}$ the variational Ricci tensor is not subject to any functional constraint. The only relationship is the customary one relating covariant and controvariant tensors through the action of the extremal (i.e., background) metric tensor ${\hat{g}}_{\mu \nu }$, for consistency with PMC. For this reason, the Lagrangian principle considered here identifies an *unconstrained* principle, to be denoted as the *unconstrained Ricci Lagrangian principle*.

Let us now calculate the Frechet derivative according to the definition (35):

(45) $$\delta S_{R}=\int_{Q^{4}}d\Omega \delta L_{R}\left(R^{\mu \nu },\alpha_{i}\right)=0.$$

This yields

(46) $${\left.\left(2\alpha_{1}R_{\mu \nu }-\alpha_{2}R{\hat{g}}_{\mu \nu }+\alpha_{3}\Lambda {\hat{g}}_{\mu \nu }\right)\right|}_{R_{\mu \nu }={\hat{R}}_{\mu \nu }}=0,$$

from which requirement of identity with EFE implies that necessarily the $\alpha_{i}$ coefficients are

(47) $$\begin{array}{ccc} \alpha_{1} & = & \alpha_{2}=\frac{1}{2}, \end{array}$$

(48) $$\begin{array}{ccc} \alpha_{3} & = & 1. \end{array}$$

In conclusion, the sought Ricci Lagrangian postulated in Equation (42) is found to be

(49) $$L_{R}\left(R^{\mu \nu },\alpha_{i}\right)=\frac{1}{2}\rho -\frac{1}{4}R^{2}+\Lambda R.$$

The notable features to highlight for this solution are:

(1) The consistency with PMC: the Ricci Lagrangian variational principle fully satisfies the requirements set by PMC, to be realized by the fact that the independent variational Lagrangian field is a 4-tensor, while the Ricci variational Lagrangian function is a 4-scalar. In addition, manifest covariance is consistently defined by the existence of a background metric tensor, with respect to which 4-tensor properties as well as raising/lowering of indices are defined.

(2) The Ricci Lagrangian principle is unconstrained, so that variations of the Ricci field are not subject to any restriction. This feature is of potential advantage for the construction of a corresponding Hamiltonian principle from the Lagrangian one by means of canonical formalism (see Ref. [14] in this respect).

## 5. Metric-Tensor Lagrangian Principles

In this section, we consider a second realization of the general formalism introduced in Section 3. This is obtained by identifying the tensor $Z^{\mu \nu }$ with the variational metric tensor $g^{\mu \nu }$. Two separate solutions can be envisaged in this respect, depending on whether the variational field is assumed to be subject to functional constraints or it remains unconstrained. One obtains therefore two distinct variational principles, to be referred to, respectively, as constrained and unconstrained metric Lagrangian principles. For completeness, we discuss the two cases separately.

### 5.1. Constrained Metric Lagrangian Principle

The first occurrence is represented by the constrained Lagrangian principle, which is at the basis of the Hilbert–Einstein (HE) principle. In this case, the variational field identifies a metric tensor subject to precise functional constraints. The goal here is to prove that such a framework is intrinsically incompatible with PMC, and therefore it fails in reproducing EFE correctly. The validity of the principle, in fact, can only be restored at the expense of violating PMC and implementing the formalism of the HE original approach.

In order to illustrate the issue, we start by setting the identification

(50) $$Z^{\mu \nu }=g^{\mu \nu },$$

where $g^{\mu \nu }$ is not only a variational field, i.e., the generalized Lagrangian coordinate, but also a metric tensor in itself. This means, in particular, that the same tensor raises/lowers tensor indices and is subject to the normalization constraint $g_{\mu \nu }g^{\mu k}=\delta_{\nu }^{k}$. The variational metric tensor therefore exhibits the same algebraic properties of the extremal (i.e., background) metric tensor. The corresponding functional class of variations identifies the constrained functional class ${\left\{g\right\}}_{C}$ given by the set

(51) $${\left\{g\right\}}_{C}\equiv \left\{\begin{array}{c} Z^{\mu \nu }\equiv g^{\mu \nu }\\ g_{\mu \nu }g^{\mu k}=\delta_{\nu }^{k}\\ {\hat{g}}_{\mu \nu }{\hat{g}}^{\mu k}=\delta_{\nu }^{k} \end{array}\right\}.$$

The evaluation of the 4-scalar *Z* according to Equation (30) demands the covariant metric tensor $g_{\mu \nu }$ in $G_{\mu \nu }$ to be variational too, so that $Z_{\mu \nu }=g_{\mu \nu }$, while $\left\{{\hat{z}}_{\mu \nu }-{\hat{Z}}_{\mu \nu }\right\}=\left\{{\hat{R}}_{\mu \nu }\right\}$. In particular, in such a framework the Ricci tensor must be regarded as an independent tensor in order to warrant consistency with PMC. The Ricci scalar becomes accordingly $R={\hat{R}}^{\alpha \beta }g_{\alpha \beta }$. Hence, we have formally that

(52) $$G_{\mu \nu }=G_{\mu \nu }\left(g_{\mu \nu },{\hat{R}}_{\mu \nu }\right),$$

and correspondingly

(53) $$Z=g^{\mu \nu }G_{\mu \nu }\left(g_{\mu \nu },{\hat{R}}_{\mu \nu }\right).$$

Explicit calculation then gives

(54) $$Z=g^{\mu \nu }{\hat{R}}_{\mu \nu }-\frac{1}{2}\left({\hat{R}}^{\alpha \beta }g_{\alpha \beta }\right)g^{\mu \nu }g_{\mu \nu }+\Lambda g^{\mu \nu }g_{\mu \nu },$$

namely after straightforward algebra

(55) $$Z=-g^{\mu \nu }{\hat{R}}_{\mu \nu }+4\Lambda ,$$

where we have made use of the normalization property of $g^{\mu \nu }$, so that for index summation $g_{\mu \nu }g^{\mu \nu }=4$.

The constrained metric Lagrangian function is correspondingly identified with

(56) $$L_{Z}\left(Z,\alpha_{i}\right)\to L_{g_{C}}=L_{g_{C}}\left(g^{\mu \nu },\alpha_{i}\right),$$

where

(57) $$L_{g_{C}}\left(g^{\mu \nu },\alpha_{i}\right)=-\alpha_{1}g^{\mu \nu }{\hat{R}}_{\mu \nu }+\alpha_{2}4\Lambda ,$$

and in this case only two numerical coefficients appear. Adopting analogous notation, the action integral becomes

(58) $$S_{g_{C}}\equiv \int_{Q^{4}}d\Omega L_{g_{C}}\left(g^{\mu \nu },\alpha_{i}\right),$$

with $L_{g_{C}}\left(g^{\mu \nu },\alpha_{i}\right)$ being given by Equation (57). The Lagrangian principle that arises is a *constrained* principle and is denoted as a *constrained metric Lagrangian principle*. If we compute the Frechet derivative according to the definition (35), we have

(59) $$\delta S_{g_{C}}=\int_{Q^{4}}d\Omega \delta L_{g_{C}}\left(g^{\mu \nu },\alpha_{i}\right)=0.$$

However, it is immediate to see that this yields

(60) $$-\alpha_{1}{\hat{R}}_{\mu \nu }=0,$$

where $\alpha_{2}$ remains indeterminate, and there is no choice of constant numerical coefficient $\alpha_{1}$ for which the previous equation can reproduce correctly the complete form of EFE. Hence, it must be concluded that necessarily the requirements set by joint validity of PMC and the variational framework represented by the constrained metric principle are incompatible. In particular, the constrained principle cannot generate the Einstein tensor equations of GR. The reason is that when the normalization condition $g_{\mu \nu }g^{\mu \nu }=4$ is assumed in the Lagrangian function, fundamental variational contributions that are quadratic in the metric tensor are lost by assumption and can no longer be recovered in the Lagrangian (57).

There is only one possible way to restore the correct validity of the variational principle. In order to recover the variational terms lost due to normalization of $g_{\mu \nu }$, it is necessary to assume that also the metric contribution to the volume element $d\Omega =d^{4}r\sqrt{-\left|g\right|}$ is variational. The route requires, however, the violation of PMC, since the dependence contained in dΩ is through the determinant $\left|g\right|$ which is not an invariant quantity by itself. Hence, we define the Lagrangian density

(61) $$L_{g_{C}}\equiv \sqrt{-\left|g\right|}L_{g_{C}},$$

which is not a 4-scalar by construction, and the corresponding action integral as

(62) $$S_{g_{C}}\equiv \int_{Q^{4}}d^{4}rL_{g_{C}}\left(g^{\mu \nu },\alpha_{i}\right).$$

The variational principle then requires

(63) $$\delta S_{g_{C}}=\int_{Q^{4}}d^{4}r\delta L_{g_{C}}\left(g^{\mu \nu },\alpha_{i}\right)=0.$$

It is immediate to verify that the Lagrangian density $L_{g_{C}}\left(g^{\mu \nu },\alpha_{i}\right)$ yields the correct form of EFE for the set of coefficients $\alpha_{1}=-1$ and $\alpha_{2}=-1/2$. Indeed, the non-tensorial factor $\sqrt{-\left|g\right|}$ replaces the quadratic terms $g_{\mu \nu }g^{\mu \nu }$, which in the constrained framework are always identically equal to four for both variational and extremal metric field tensors $g_{\mu \nu }$. We notice that:

(1) The simultaneous validity of conditions set by PMC and the constrained metric variational principle are incompatible. The correctness of the constrained variational principle can be restored only under violation of PMC, namely by treating the Lagrangian density $L_{g_{C}}$, which is not an invariant quantity, instead of the 4-scalar Lagrangian $L_{g_{C}}$. This amounts to effectively treating a constrained variational principle not preserving the space-time volume element. According to the definitions given in Ref. [23], this realizes an asynchronous variational principle.

(2) The solution reproduces the HE variational principle. In fact, as recalled in Section 2, for the derivation of EFE in the framework of the HE principle, only the explicit dependences on $g^{\mu \nu }$ contained in the constrained Lagrangian effectively matter. Instead, the implicit dependences carried by the Christoffel symbols in the Ricci tensor do not contribute and, if present, should be suitably ruled out with appropriate boundary conditions. Remarkably, these contributions determine the non-standard feature of the HE Lagrangian, namely its so-called non-first-order character. In turn, the present derivation provides a physical explanation on why such dependences are not essential for the correct derivation of EFE.

(3) The constrained variational principle (63) violates manifest covariance as well as the fundamental gauge-invariace properties characteristic of variational principles for continuum fields of classical and relativistic mechanics. This feature is emphasized by the fact that the 4-volume element must be variational, a feature that is peculiar of HE theory.

### 5.2. Unconstrained Metric Lagrangian Principle

The second occurrence is realized by the unconstrained Lagrangian principle, which provides the theoretical framework for the synchronous variational principle introduced in Section 2. In this case, the variational field is a second-order symmetric tensor denoted with $g_{\mu \nu }$, which does not identify a metric tensor, and for this reason it is not subject to functional constraints. In contrast, the metric tensor represents a background space-time metric tensor to be denoted by the symbol ${\hat{g}}_{\mu \nu }$, which raises/lowers tensor indices and is subject to the normalization constraint ${\hat{g}}_{\mu \nu }{\hat{g}}^{\mu k}=\delta_{\nu }^{k}$. By definition, in the variational principle, ${\hat{g}}_{\mu \nu }$ remains distinguished from $g_{\mu \nu }$. The goal in this case is to prove that such a framework permits the realization of a variational theory in agreement with PMC which correctly reproduces EFE.

In order to set the issue on a mathematical basis, we start by setting the identification

(64) $$Z^{\mu \nu }=g^{\mu \nu },$$

where $g^{\mu \nu }$ is a variational field that does not exhibit the same algebraic properties of the extremal (i.e., background) metric tensor, and therefore we require that $g_{\mu \nu }\neq {\hat{g}}_{\mu \nu }$ in the variational principle. On the other hand, the two tensors are required to coincide in EFE, namely ${\left.g_{\mu \nu }\right|}_{extr}=$${\hat{g}}_{\mu \nu }$. The corresponding functional class of variations identifies the unconstrained functional class ${\left\{g\right\}}_{U}$ defined by the set

(65) $${\left\{g\right\}}_{U}\equiv \left\{\begin{array}{c} Z^{\mu \nu }\equiv g^{\mu \nu }\neq {\hat{g}}^{\mu \nu }\\ g_{\mu \nu }g^{\mu k}\neq \delta_{\nu }^{k}\\ {\hat{g}}_{\mu \nu }{\hat{g}}^{\mu k}=\delta_{\nu }^{k}\\ g^{\mu \nu }={\hat{g}}^{\mu \alpha }{\hat{g}}^{\nu \beta }g_{\alpha \beta }\\ {\left.g_{\mu \nu }\right|}_{extr}={\hat{g}}_{\mu \nu } \end{array}\right\}.$$

The evaluation of the 4-scalar *Z* according to Equation (30) demands again the covariant metric tensor $g_{\mu \nu }$ in $G_{\mu \nu }$ to be variational too, so that $Z_{\mu \nu }=g_{\mu \nu }$, while $\left\{{\hat{z}}_{\mu \nu }-{\hat{Z}}_{\mu \nu }\right\}=\left\{{\hat{R}}_{\mu \nu }\right\}$. For the same reason, consistent with PMC, also in such a framework the Ricci tensor must be regarded as an independent tensor. The Ricci scalar becomes accordingly $R={\hat{R}}^{\alpha \beta }g_{\alpha \beta }$. Hence, we have formally that

(66) $$G_{\mu \nu }=G_{\mu \nu }\left(g_{\mu \nu },{\hat{R}}_{\mu \nu }\right),$$

and correspondingly

(67) $$Z=g^{\mu \nu }G_{\mu \nu }\left(g_{\mu \nu },{\hat{R}}_{\mu \nu }\right).$$

Explicit calculation then gives

(68) $$Z=g^{\mu \nu }{\hat{R}}_{\mu \nu }-\frac{1}{2}\left({\hat{R}}^{\alpha \beta }g_{\alpha \beta }\right)g^{\mu \nu }g_{\mu \nu }+\Lambda g^{\mu \nu }g_{\mu \nu },$$

where now the quadratic terms proportional to $g^{\mu \nu }g_{\mu \nu }$ are retained according to the prescriptions set by the functional class ${\left\{g\right\}}_{U}$.

The unconstrained metric Lagrangian function is correspondingly identified with

(69) $$L_{Z}\left(Z,\alpha_{i}\right)\to L_{g_{U}}=L_{g_{U}}\left(g^{\mu \nu },\alpha_{i}\right),$$

where

(70) $$L_{gU}\left(g^{\mu \nu },\alpha_{i}\right)=\alpha_{1}g^{\mu \nu }{\hat{R}}_{\mu \nu }-\alpha_{2}\frac{1}{2}\left({\hat{R}}^{\alpha \beta }g_{\alpha \beta }\right)g^{\mu \nu }g_{\mu \nu }+\alpha_{3}\Lambda g^{\mu \nu }g_{\mu \nu }.$$

Adopting analogous notation, the action integral becomes

(71) $$S_{g_{U}}\equiv \int_{Q^{4}}d\Omega L_{g_{U}}\left(g^{\mu \nu },\alpha_{i}\right),$$

with $L_{g_{U}}\left(g^{\mu \nu },\alpha_{i}\right)$ being given by Equation (70), and here the volume element depends on the background metric tensor, namely $d\Omega =d^{4}r\sqrt{-\left|\hat{g}\right|}\equiv d\hat{\Omega }$. The Lagrangian principle then identifies an *unconstrained* principle, which is denoted as the *unconstrained metric Lagrangian principle*.

Let us now calculate the Frechet derivative according to the definition (35):

(72) $$\delta S_{g_{U}}=\int_{Q^{4}}d\Omega \delta L_{g_{U}}\left(g^{\mu \nu },\alpha_{i}\right)=0.$$

This yields after straightforward algebra

(73) $${\left.\left(\left(\alpha_{1}-2\alpha_{2}\right){\hat{R}}_{\mu \nu }+\left(2\alpha_{3}\Lambda -\alpha_{2}{\hat{R}}^{\alpha \beta }g_{\alpha \beta }\right)g_{\mu \nu }\right)\right|}_{g_{\mu \nu }={\hat{g}}_{\mu \nu }}=0,$$

from which the requirement of identity with EFE implies that necessarily the $\alpha_{i}$ coefficients are

(74) $$\begin{array}{ccc} \alpha_{1} & = & 2, \end{array}$$

(75) $$\begin{array}{ccc} \alpha_{2} & = & \alpha_{3}=\frac{1}{2}. \end{array}$$

Replacing the solution in Equation (70) yields the unconstrained metric Lagrangian in the form

(76) $$L_{gU}\left(g^{\mu \nu },\alpha_{i}\right)=2g^{\mu \nu }{\hat{R}}_{\mu \nu }-\frac{1}{4}\left({\hat{R}}^{\alpha \beta }g_{\alpha \beta }\right)g^{\mu \nu }g_{\mu \nu }+\frac{1}{2}\Lambda g^{\mu \nu }g_{\mu \nu }.$$

Rearranging the terms and changing summed indices gives the compact representation

(77) $$L_{gU}\left(g^{\mu \nu },\alpha_{i}\right)=\left(2-\frac{1}{4}g^{\alpha \beta }g_{\alpha \beta }\right)g^{\mu \nu }{\hat{R}}_{\mu \nu }+\frac{1}{2}\Lambda g^{\mu \nu }g_{\mu \nu }.$$

In order to highlight the connection with the synchronous variational principle recalled in Section 2, we can then define the variational weight factor $h\left(g\right)\equiv h\left(g_{\mu \nu },{\hat{g}}_{\mu \nu }\right)$ as

(78) $$h\left(g_{\mu \nu },{\hat{g}}_{\mu \nu }\right)=2-\frac{1}{4}g^{\alpha \beta }g_{\alpha \beta },$$

so that we finally obtain the unconstrained or synchronous metric Lagrangian

(79) $$L_{gU}\left(g^{\mu \nu },\alpha_{i}\right)=h\left(g\right)g^{\mu \nu }{\hat{R}}_{\mu \nu }+\frac{1}{2}\Lambda g^{\mu \nu }g_{\mu \nu }.$$

Equivalently, we can also write it in the more compact form

(80) $$L_{gU}\left(g^{\mu \nu },\alpha_{i}\right)=h\left(g\right)\left(g^{\mu \nu }{\hat{R}}_{\mu \nu }-2\Lambda \right),$$

which differs from Equation (79) only for a constant numerical factor.

The following comments are in order:

(1) The unconstrained metric Lagrangian principle and its realization in terms of synchronous variational principle consistently satisfy PMC and the gauge-invariance properties holding for classical variational principles of continuum fields.

(2) In the unconstrained framework, the invariant 4-volume element is treated as extremal, namely as a function of the background metric tensor. This feature justifies the use of the word “synchronous” to identify the principle, in analogy with a similar feature arising for integration-time line-element in variational principles of classical mechanics. This mathematical property of the variational principle marks a point of connection with standard Lagrangian principles holding in continuum field theory, where variational behavior pertains to the field Lagrangian function and not to the differential integration element of the action integral. Furthermore, under the same assumption, the variational Lagrangian remains necessarily identified with a 4-scalar, and not with a scalar density, so that PMC remains automatically satisfied.

(3) Based on the unconstrained variational theory developed above, the construction of the Lagrangian (79) characteristic of the synchronous variational principle allows one to determine the unique form of the variational factor $h\left(g\right)$ and to point out its physical meaning. The choice of the form of $h\left(g\right)$ is not arbitrary or a matter of fact, but follows from precise physical and mathematical requirements at the basis of the unconstrained Lagrangian principle. The role of $h\left(g\right)$ is to warrant the derivation of the correct form of EFE together with the simultaneous validity of PMC. It replaces the variational contributions that in the constrained principle arise from variation of $\sqrt{-\left|g\right|}$ in the volume element of integration, but without violating PMC. Finally, as a matter of consistency, the extremal value of $h\left(g\right)$ is such that $h\left(\hat{g}\right)=1$.

(4) In the unconstrained picture, the physical meaning of $g_{\mu \nu }$ and ${\hat{g}}_{\mu \nu }$ remains distinguished. Thus, ${\hat{g}}_{\mu \nu }$ is a geometric tensor which raises/lowers tensor indices, defines the integration 4-volume element, the covariant derivatives as well as the Ricci tensor of the background space-time. Instead, the variational tensor $g_{\mu \nu }$ plays the role of a physical tensor associated with the gravitational field, for which “kinetic”, “potential” and “coupling” terms can be assigned in the variational principle for the derivation of the corresponding dynamical evolution equations. Remarkably, consistent with the principles of GR, the physical and geometrical properties of space-time are realized in terms of a single tensor in the extremal field equations, namely EFE, by means of the identification ${\left.g_{\mu \nu }\right|}_{extr}={\hat{g}}_{\mu \nu }$.

## 6. Tangent 4-Vectors Lagrangian Principles

In this section, we explore further the validity of the general formalism introduced above for the variational formulation of EFE and its realization in terms of constrained and unconstrained principles. The outcome of this analysis will be useful to draw conclusions about the physical relevance and correctness of unconstrained Lagrangian principles with respect to the constrained ones. To this aim, we consider two additional GR symmetric tensors that can be used to construct corresponding admissible Lagrangian functions. The first one is associated with the 4-velocity ${\hat{u}}^{\mu }$ tangent to subluminal geodesic curves of space-time. Thus, we assume that in the background space-time there exists a geodesic curve whose tangent unit 4-vector ${\hat{u}}^{\mu }\equiv \frac{dr^{\mu }}{ds}$, with *s* denoting the geodetic proper-time, satisfies the normalization condition

(81) $${\hat{g}}_{\mu \nu }{\hat{u}}^{\mu }{\hat{u}}^{\nu }=1.$$

The symmetric dyadic tensor ${\hat{u}}^{\mu }{\hat{u}}^{\nu }$ can then represent a plausible tensor for our purpose. Similarly, the second tensor of interest is associated with the null wave 4-vector ${\hat{k}}^{\mu }\equiv \frac{dr^{\mu }}{d\lambda }$, with dλ denoting a suitable parameter varying along a light-ray. The latter 4-vector by definition satisfies the 4-scalar identity (orthogonality condition)

(82) $${\hat{g}}_{\mu \nu }{\hat{k}}^{\mu }{\hat{k}}^{\nu }=0,$$

so that also the symmetric tensor ${\hat{k}}^{\mu }{\hat{k}}^{\nu }$ provides an admissible choice for the present task. Therefore, we can consider separately the role of the two tensors ${\hat{u}}^{\mu }{\hat{u}}^{\nu }$ and ${\hat{k}}^{\mu }{\hat{k}}^{\nu }$, respectively, for the validity of constrained and unconstrained Lagrangian principles for EFE.

### 6.1. Constrained Tangent 4-Vectors Lagrangian Principles

We start again analyzing the case of constrained Lagrangian principles for the two sets of variational tensors $u^{\mu }u^{\nu }$ and $k^{\mu }k^{\nu }$, which are accordingly taken to be subject to precise functional constraints. The goal is to prove that such a framework fails to reproduce EFE as extremal equations, in formal analogy with the case of the constrained metric Lagrangian principle. To proceed with the proof, we start by making the identification

(83) $$Z^{\mu \nu }=u^{\mu }u^{\nu },$$

where $u^{\mu }u^{\nu }$ represents the variational tensor. The corresponding functional class of variations identifies the constrained functional class ${\left\{u\right\}}_{C}$ given by the set

(84) $${\left\{u\right\}}_{C}\equiv \left\{\begin{array}{c} Z^{\mu \nu }\equiv u^{\mu }u^{\nu }\\ {\hat{g}}_{\mu \nu }u^{\mu }u^{\nu }=1\\ {\hat{g}}_{\mu \nu }{\hat{u}}^{\mu }{\hat{u}}^{\nu }=1 \end{array}\right\}.$$

This means that both the extremal and the variational 4-vectors $u^{\mu }$ and ${\hat{u}}^{\mu }$ are subject to the same normalization condition. We further notice that the tensor ${\hat{u}}^{\mu }{\hat{u}}^{\nu }$ does not belong to the set ${\hat{z}}_{\mu \nu }$, so that we can write

(85) $$G_{\mu \nu }=G_{\mu \nu }\left({\hat{g}}_{\mu \nu },{\hat{R}}_{\mu \nu }\right),$$

and correspondingly

(86) $$Z=u^{\mu }u^{\nu }G_{\mu \nu }\left({\hat{g}}_{\mu \nu },{\hat{R}}_{\mu \nu }\right).$$

Explicit calculation then gives

(87) $$Z=u^{\mu }u^{\nu }{\hat{R}}_{\mu \nu }-\frac{1}{2}\hat{R}u^{\mu }u^{\nu }{\hat{g}}_{\mu \nu }+\Lambda u^{\mu }u^{\nu }{\hat{g}}_{\mu \nu },$$

where $\hat{R}={\hat{R}}^{\alpha \beta }{\hat{g}}_{\alpha \beta }$, so that according to ${\left\{u\right\}}_{C}$ one obtains

(88) $$Z=u^{\mu }u^{\nu }{\hat{R}}_{\mu \nu }-\frac{1}{2}\hat{R}+\Lambda .$$

The constrained Lagrangian function is correspondingly identified with

(89) $$L_{Z}\left(Z,\alpha_{i}\right)\to L_{u_{C}}=L_{u_{C}}\left(u^{\mu }u^{\nu },\alpha_{i}\right),$$

where

(90) $$L_{u_{C}}\left(u^{\mu }u^{\nu },\alpha_{i}\right)=\alpha_{1}u^{\mu }u^{\nu }{\hat{R}}_{\mu \nu }-\alpha_{2}\frac{1}{2}\hat{R}+\alpha_{3}\Lambda ,$$

while the action integral becomes

(91) $$S_{u_{C}}\equiv \int_{Q^{4}}d\Omega L_{u_{C}}\left(u^{\mu }u^{\nu },\alpha_{i}\right),$$

with $L_{u_{C}}\left(u^{\mu }u^{\nu },\alpha_{i}\right)$ being given by Equation (90). Application of the Frechet derivative according to the definition (35) gives

(92) $$\delta S_{u_{C}}=\int_{Q^{4}}d\Omega \delta L_{u_{C}}\left(u^{\mu }u^{\nu },\alpha_{i}\right)=0,$$

which determines the *constrained unit* 4-*vector Lagrangian principle*, with $u^{\mu }u^{\nu }$ being the variational tensor field. However, it is immediate to see that this yields

(93) $$\alpha_{1}{\hat{R}}_{\mu \nu }=0,$$

where both $\alpha_{2}$ and $\alpha_{3}$ remain indeterminate and there is no choice of constant numerical coefficient $\alpha_{1}$ for which the previous equation can reproduce correctly the complete form of EFE. Hence, it must be concluded that in such a case the constrained variational principle fails.

A similar conclusion can be inferred in case one would pick up the tensor $Z^{\mu \nu }=k^{\mu }k^{\nu }$ as variational field. The corresponding constrained functional class of variations ${\left\{k\right\}}_{C}$ is given by the set

(94) $${\left\{k\right\}}_{C}\equiv \left\{\begin{array}{c} Z^{\mu \nu }\equiv k^{\mu }k^{\nu }\\ {\hat{g}}_{\mu \nu }k^{\mu }k^{\nu }=0\\ {\hat{g}}_{\mu \nu }{\hat{k}}^{\mu }{\hat{k}}^{\nu }=0 \end{array}\right\}.$$

Explicit calculation of the 4-scalar *Z* then gives

(95) $$Z=k^{\mu }k^{\nu }{\hat{R}}_{\mu \nu }-\frac{1}{2}\hat{R}k^{\mu }k^{\nu }{\hat{g}}_{\mu \nu }+\Lambda k^{\mu }k^{\nu }{\hat{g}}_{\mu \nu },$$

so that according to ${\left\{k\right\}}_{C}$ two terms disappear, and one obtains simply

(96) $$Z=k^{\mu }k^{\nu }{\hat{R}}_{\mu \nu }.$$

The constrained metric Lagrangian function is correspondingly identified with

(97) $$L_{Z}\left(Z,\alpha_{i}\right)\to L_{k_{C}}=L_{k_{C}}\left(k^{\mu }k^{\nu },\alpha_{i}\right),$$

where

(98) $$L_{k_{C}}\left(k^{\mu }k^{\nu },\alpha_{i}\right)=\alpha_{1}k^{\mu }k^{\nu }{\hat{R}}_{\mu \nu },$$

while the action integral becomes

(99) $$S_{k_{C}}\equiv \int_{Q^{4}}d\Omega L_{k_{C}}\left(k^{\mu }k^{\nu },\alpha_{i}\right),$$

with $L_{k_{C}}\left(k^{\mu }k^{\nu },\alpha_{i}\right)$ being given by Equation (98). The corresponding *constrained null* 4-*vector Lagrangian principle* gives

(100) $$\delta S_{k_{C}}=\int_{Q^{4}}d\Omega \delta L_{k_{C}}\left(k^{\mu }k^{\nu },\alpha_{i}\right)=0.$$

Again, it is immediate to see that under the present assumptions this yields

(101) $$\alpha_{1}{\hat{R}}_{\mu \nu }=0,$$

where both $\alpha_{2}$ and $\alpha_{3}$ remain indeterminate, and there is no choice of constant numerical coefficient $\alpha_{1}$ for which the previous equation can reproduce correctly the complete form of EFE. Hence, it must be concluded that also in such a case the constrained variational principle fails.

We notice that the situation depicted here is exactly analogous to the case of constrained metric Lagrangian principle. The negative output is a consequence of the assumption of validity of normalization/orthogonality conditions applying to either $u^{\mu }u^{\nu }$ or $k^{\mu }k^{\nu }$, both for their variational and extremal realizations. However, while in the case of the metric principle the problem could be circumvented by assuming the volume element to be variational too, thus violating PMC, in the present case an alternative of this type is no longer possible. On the other hand, the choices $Z^{\mu \nu }=u^{\mu }u^{\nu }$ or $Z^{\mu \nu }=k^{\mu }k^{\nu }$ are admissible ones, which means that the only point of weakness in such a framework can only be the same concept of constrained principle associated with PMC. Namely, the fact that, in the constrained functional classes, the variational tensor fields possess the same normalization/orthogonality conditions characteristic of their corresponding extremal fields. It is evident at this stage that this kind of assumption is critical and must be abandoned, considering also the fact that there is no compelling physical reason supporting its adoption.

### 6.2. Unconstrained Tangent 4-Vectors Lagrangian Principle

As a final step, we evaluate the unconstrained variational framework for the two sets of variational tensors $u^{\mu }u^{\nu }$ and $k^{\mu }k^{\nu }$. The goal here is to prove that this represents the correct approach for the establishment of the variational theory for EFE that satisfies PMC. As shown below, in the unconstrained formalism the two tensors $u^{\mu }u^{\nu }$ and $k^{\mu }k^{\nu }$ behave the same in the variational principle. We start again by identifying $Z^{\mu \nu }=u^{\mu }u^{\nu }$, where $u^{\mu }u^{\nu }$ is the variational tensor, not to be subject to functional constraints.

The corresponding unconstrained functional class of variations ${\left\{u\right\}}_{U}$ is given by

(102) $${\left\{u\right\}}_{U}\equiv \left\{\begin{array}{c} Z^{\mu \nu }\equiv u^{\mu }u^{\nu }\\ {\hat{g}}_{\mu \nu }u^{\mu }u^{\nu }\neq 1\\ {\hat{g}}_{\mu \nu }{\hat{u}}^{\mu }{\hat{u}}^{\nu }=1 \end{array}\right\},$$

where $u^{\mu }$ and ${\hat{u}}^{\mu }$ are the variational and the extremal 4-vectors, respectively. Since $G_{\mu \nu }=G_{\mu \nu }\left({\hat{g}}_{\mu \nu },{\hat{R}}_{\mu \nu }\right)$, we have that

(103) $$Z=u^{\mu }u^{\nu }G_{\mu \nu }\left({\hat{g}}_{\mu \nu },{\hat{R}}_{\mu \nu }\right).$$

Explicit calculation for $u^{\mu }u^{\nu }\in {\left\{u\right\}}_{U}$ then gives

(104) $$Z=u^{\mu }u^{\nu }{\hat{R}}_{\mu \nu }-\frac{1}{2}\hat{R}u^{\mu }u^{\nu }{\hat{g}}_{\mu \nu }+\Lambda u^{\mu }u^{\nu }{\hat{g}}_{\mu \nu },$$

where $\hat{R}={\hat{R}}^{\alpha \beta }{\hat{g}}_{\alpha \beta }$. The unconstrained Lagrangian function is correspondingly identified with

(105) $$L_{Z}\left(Z,\alpha_{i}\right)\to L_{u_{U}}=L_{u_{U}}\left(u^{\mu }u^{\nu },\alpha_{i}\right),$$

where

(106) $$L_{u_{U}}\left(u^{\mu }u^{\nu },\alpha_{i}\right)=\alpha_{1}u^{\mu }u^{\nu }{\hat{R}}_{\mu \nu }-\alpha_{2}\frac{1}{2}\hat{R}u^{\mu }u^{\nu }{\hat{g}}_{\mu \nu }+\alpha_{3}\Lambda u^{\mu }u^{\nu }{\hat{g}}_{\mu \nu },$$

while the action integral becomes

(107) $$S_{u_{U}}\equiv \int_{Q^{4}}d\Omega L_{u_{U}}\left(u^{\mu }u^{\nu },\alpha_{i}\right),$$

with $L_{u_{U}}\left(u^{\mu }u^{\nu },\alpha_{i}\right)$ being given by Equation (106). The *unconstrained unit* 4-*vector Lagrangian principle* is then written as

(108) $$\delta S_{u_{U}}=\int_{Q^{4}}d\Omega \delta L_{u_{U}}\left(u^{\mu }u^{\nu },\alpha_{i}\right)=0.$$

Thanks to the linear dependence of $L_{u_{U}}\left(u^{\mu }u^{\nu },\alpha_{i}\right)$ on $u^{\mu }u^{\nu }$, straightforward algebra yields

(109) $$\alpha_{1}{\hat{R}}_{\mu \nu }-\alpha_{2}\frac{1}{2}\hat{R}{\hat{g}}_{\mu \nu }+\alpha_{3}\Lambda {\hat{g}}_{\mu \nu }=0,$$

from which requirement of identity with EFE implies that necessarily the $\alpha_{i}$ coefficients are

(110) $$\alpha_{1}=\alpha_{2}=\alpha_{3}=1.$$

Replacing the solution in Equation (106) gives the final representation of the unconstrained Lagrangian in the form

(111) $$L_{u_{U}}\left(u^{\mu }u^{\nu },\alpha_{i}\right)=u^{\mu }u^{\nu }{\hat{R}}_{\mu \nu }-\frac{1}{2}\hat{R}u^{\mu }u^{\nu }{\hat{g}}_{\mu \nu }+\Lambda u^{\mu }u^{\nu }{\hat{g}}_{\mu \nu }.$$

Finally, the case of null 4-vector is formally the same, apart from the change of normalization condition with orthogonality condition in the functional class. Briefly, if we now set $Z^{\mu \nu }=k^{\mu }k^{\nu }$, the corresponding unconstrained functional class of variations ${\left\{k\right\}}_{U}$ for the null 4-vector is given by

(112) $${\left\{k\right\}}_{U}\equiv \left\{\begin{array}{c} Z^{\mu \nu }\equiv k^{\mu }k^{\nu }\\ {\hat{g}}_{\mu \nu }k^{\mu }k^{\nu }\neq 0\\ {\hat{g}}_{\mu \nu }{\hat{k}}^{\mu }{\hat{k}}^{\nu }=0 \end{array}\right\}.$$

The unconstrained Lagrangian function is correspondingly identified with

(113) $$L_{Z}\left(Z,\alpha_{i}\right)\to L_{k_{U}}=L_{k_{U}}\left(k^{\mu }k^{\nu },\alpha_{i}\right),$$

while the action integral becomes

(114) $$S_{k_{U}}\equiv \int_{Q^{4}}d\Omega L_{k_{U}}\left(k^{\mu }k^{\nu },\alpha_{i}\right).$$

The *unconstrained null* 4-*vector Lagrangian principle* then is written as

(115) $$\delta S_{k_{U}}=\int_{Q^{4}}d\Omega \delta L_{k_{U}}\left(k^{\mu }k^{\nu },\alpha_{i}\right)=0.$$

Algebraic calculation analogous to that carried out for $u^{\mu }u^{\nu }$ then gives in conclusion the representation of the unconstrained Lagrangian in the form

(116) $$L_{k_{U}}\left(k^{\mu }k^{\nu },\alpha_{i}\right)=k^{\mu }k^{\nu }{\hat{R}}_{\mu \nu }-\frac{1}{2}\hat{R}k^{\mu }k^{\nu }{\hat{g}}_{\mu \nu }+\Lambda k^{\mu }k^{\nu }{\hat{g}}_{\mu \nu }.$$

We notice that, contrary to the constrained setting, the unconstrained framework works because no normalization conditions are imposed a priori on the variational tensor fields. The latter conditions are required to be satisfied only by the extremal fields, i.e., the physically observable fields, and not by the virtual variational fields. The positive outcome of the unconstrained approach against the failure of the constrained one also for the set of variational tensors $u^{\mu }u^{\nu }$ and $k^{\mu }k^{\nu }$ marks a remarkable point of contact with the theory of metric Lagrangian principles. In particular, both derivations exclude the possibility to obtain constrained variational principles that satisfy PMC and reproduce EFE. Therefore, according to the present theory and the mathematical proofs reported above, only the unconstrained variational principles possess the correct properties for a consistent manifestly covariant variational formulation of EFE.

## 7. Treatment of External Sources

In previous sections, we have focused on development of variational principles corresponding to vacuum EFE, namely in the absence of external sources. This is obtained by setting in Equation (16) ${\hat{T}}_{\mu \nu }=0$. It is necessary now to establish how the treatment of external sources can be dealt with in the frameworks of constrained and unconstrained variational principles. The goal is the proof that the novel unconstrained theory is compatible with the existence of source fields, to be described either in terms of corresponding Lagrangian functions or through their stress-energy tensor. In particular, in this respect the following two possibilities can be envisaged:

(1) *Stress-energy tensor of source fields*—The first realization is achieved by assigning directly the symmetric stress-energy tensor ${\hat{T}}_{\mu \nu }$ of the source field, to be assumed known and prescribed. For the validity of the general formalism introduced in Section 2, the tensor ${\hat{T}}_{\mu \nu }$ must be regarded as an independent tensor not depending on variational fields. Hence, possible functional dependences carried by ${\hat{T}}_{\mu \nu }$ on variational fields (such as for example $g_{\mu \nu }$ in the metric Lagrangian principle) must be excluded in the present formalism. For this reason, we retain the notation of the stress-energy tensor with the hat recalling it represents a given background field. Then, given these premises, the inclusion of ${\hat{T}}_{\mu \nu }$ in the formalism for the construction of variational principles proceeds in the same way as for the tensor $G_{\mu \nu }$. In particular, one takes the index saturation with the tensor $Z^{\mu \nu }$ corresponding to each case discussed above, and simply includes the term $\kappa Z^{\mu \nu }{\hat{T}}_{\mu \nu }$ in the Lagrangian function $L_{Z}\left(Z,\alpha_{i}\right)$. Given the linearity of the product with $Z^{\mu \nu }$, there is no need to multiply this term by any coefficient $\alpha_{i}$. The variational calculus with respect to the variational field $Z^{\mu \nu }$ then delivers the correct form of non-vacuum EFE, preserving the validity of PMC. The conclusions drawn above on the validity of constrained and unconstrained Lagrangian principles remain unaltered. Thus, in particular, all unconstrained principles (e.g., the Ricci and metric Lagrangian principles) admit this type of treatment of external source fields and they warrant the correct derivation of EFE. Instead, the constrained principles must be excluded among available theories, also with the inclusion of stress-energy tensor contributions, since they intrinsically fail to recover EFE as proved above. Finally, as a side comment, we notice that unconstrained Lagrangian principles depending directly on the stress-energy tensor ${\hat{T}}_{\mu \nu }$ can provide a convenient theoretical framework for the treatment of external source fields. This type of dependence in fact can be an advantage in cases in which the source Lagrangian function of the same fields is missing or unknown, while nevertheless the tensor ${\hat{T}}_{\mu \nu }$ can be obtained, e.g., through symmetry or conservation law properties.

(2) *Lagrangian function of source fields*—The second type of possible realization is the customary literature one achieved by assigning the Lagrangian function $L_{F}$ of the source fields. However, this approach can only apply in the case of metric Lagrangian functions. More precisely, it represents the only viable formulation for the constrained theory exemplified by the asynchronous variational principle. Instead, in the case of the unconstrained theory realized by the synchronous variational principle it can work in alternative to the stress-energy tensor formalism. In both cases, the functional dependences carried by $L_{F}$ in terms of metric tensor must be regarded as variational. It is immediate to prove the validity of the Lagrangian function approach in the two settings. In fact, for the asynchronous principle one must consider the variational Lagrangian density $L_{F}\equiv \sqrt{-\left|g\right|}L_{F}\left(g_{\mu \nu }\right)$, which again is not a 4-scalar and therefore violates PMC. Instead, for the unconstrained synchronous approach it is sufficient to consider the 4-scalar $h\left(g\right)L_{F}\left(g_{\mu \nu }\right)$, since the factor $h\left(g\right)$ replaces the contribution carried by $\sqrt{-\left|g\right|}$, preserving PMC.

From this analysis the superiority of the unconstrained metric Lagrangian principle over the constrained one emerges clearly. In fact, the unconstrained metric Lagrangian principle realized by the synchronous principle represents the only method among those discussed above for which either the source stress-energy or source Lagrangian function theories are allowed and can apply. This promotes the novel synchronous metric principle to be a complete and physically relevant framework for investigating the variational theory of EFE.

## 8. Physical Relevance of Unconstrained Principles

In this section, we discuss the physical relevance of the theory of unconstrained Lagrangian principles for EFE. The focus is on the relationship of the theory with an analogous formulation holding in classical mechanics and the implication for the formulation of classical Hamiltonian and quantum gravitational theories. More precisely, the following issues are considered as application:

(1) Comparison with the theory of constrained and unconstrained variational principles in classical mechanics.

(2) Unconstrained Lagrangian principles as the natural setting for the formulation of unconstrained Hamiltonian principles.

### 8.1. Unconstrained Variational Principles in Classical Mechanics

In this section, we point out a relevant connection existing between the theory of constrained and unconstrained Lagrangian principles for EFE and an analogous distinction arising in classical relativistic mechanics for single point-particle Lagrangian dynamics. In particular, this involves the realization of constrained and unconstrained principles in terms of asynchronous and synchronous Lagrangian theories, respectively, in the two settings, namely for EFE and classical mechanics. To start with, we denote by $r^{\mu }\left(s\right)$ the Lagrangian world-line trajectory of a charged point particle with rest mass $m_{o}$, charge $q_{o}$ and proper time *s*, so that the corresponding 4-velocity is $u^{\mu }\left(s\right)=\frac{dr^{\mu }\left(s\right)}{ds}$, while

(117) $$ds^{2}=g_{\mu \nu }\left(r\left(s\right)\right)dr^{\nu }\left(s\right)dr^{\mu }\left(s\right).$$

Here, the metric tensor $g_{\mu \nu }\left(s\right)\equiv g_{\mu \nu }\left(r\left(s\right)\right)$ and the Faraday tensor $F_{\mu \nu }=\partial_{\mu }A_{\nu }-\partial_{\nu }A_{\mu }$ of the external EM fields, with $A_{\mu }\left(s\right)\equiv A_{\mu }\left(r\left(s\right)\right)$, are considered prescribed functions of *r*, namely extremal fields, where we omit for brevity in this section the hatted notation.

We consider first the definition of asynchronous principle reported for example in Ref. [10]. This represents the most commonly known version of variational theory in classical mechanics. The action functional in this case is identified with

(118) $$S_{p_{A}}\left(r\right)=-\int_{s_{1}}^{s_{2}}ds\left(g_{\mu \nu }\left(s\right)\frac{dr^{\nu }\left(s\right)}{ds}+qA_{\mu }\left(s\right)\right)\frac{dr^{\mu }\left(s\right)}{ds},$$

where $q\equiv \frac{q_{o}}{m_{o}c^{2}}$ is the normalized charge and $s_{1}$ and $s_{2}$ are fixed boundary values. In the functional $S_{p_{A}}\left(r\right)$, the function $r^{\mu }\left(s\right)$ is assumed to belong to the asynchronous functional class:

(119) $${\left\{r^{\mu }\right\}}_{A}=\left\{\begin{array}{c} r^{\mu }\left(s\right)\in C^{2}\left(R\right)\\ \delta \left(ds\right)\neq 0\\ r^{\mu }\left(s_{k}\right)=r_{k}^{\mu },\;k=1,2 \end{array}\right\},$$

where necessarily

(120) $$\delta \left(ds\right)=\delta \left(\sqrt{g_{\mu \nu }\left(s\right)dr^{\nu }\left(s\right)dr^{\mu }\left(s\right)}\right),$$

and δ denotes again the Frechet derivative. The asynchronous Hamilton variational principle follows from the variational equation

(121) $$S_{p_{A}}\left(r\right)\equiv {\left.\frac{d}{d\alpha }\Psi \left(\alpha \right)\right|}_{\alpha =0}=0,$$

to hold for arbitrary displacements $\delta r^{\mu }\left(s\right)$. Here, Ψ(α) is the smooth real function $\Psi \left(\alpha \right)=S_{p_{A}}\left(r+\alpha \delta r\right)$, being $\alpha \in \left[-1,1\right]$ to be considered independent of r(s) and *s*. The corresponding Euler–Lagrange equation are found to be

(122) $$\frac{\delta S_{p_{A}}\left(r\right)}{\delta r^{\mu }\left(s\right)}\equiv g_{\mu \nu }\frac{D}{Ds}\frac{dr^{\nu }\left(s\right)}{ds}-qF_{\mu \nu }\frac{dr^{\nu }\left(s\right)}{ds}=0.$$

However, also in classical mechanics, it is possible to introduce a corresponding synchronous variational principle [9]. In this case, the functional is expressed in terms of superabundant variables $r^{\mu }\left(s\right)$ and $u^{\mu }\left(s\right)$ as

(123) $$S_{p_{S}}\left(r,u\right)=-\int_{s_{1}}^{s_{2}}dsL_{p_{S}}\left(r\left(s\right),\frac{dr\left(s\right)}{ds},u\left(s\right)\right),$$

where $L_{p_{S}}\equiv L_{p_{S}}\left(r\left(s\right),\frac{dr\left(s\right)}{ds},u\left(s\right)\right)$ is the 4-scalar Lagrangian function

$$L_{p_{S}}\equiv \left(u_{\mu }\left(s\right)+qA_{\mu }\left(s\right)\right)\frac{dr^{\mu }\left(s\right)}{ds}-\frac{1}{2}u^{\mu }\left(s\right)u_{\mu }\left(s\right),$$

which is linear in $\frac{dr\left(s\right)}{ds}$. In addition, the functions $r^{\mu }\left(s\right)$ and $u^{\mu }\left(s\right)$ are required to belong to the synchronous functional class defined as

(124) $${\left\{r^{\mu },u^{\mu }\right\}}_{S}=\left\{\begin{array}{c} r^{\mu }\left(s\right),u^{\mu }\left(s\right)\in C^{2}\left(R\right)\\ \delta \left(ds\right)=0\\ u^{\mu }\left(s\right)u_{\mu }\left(s\right)\neq 1\\ r^{\mu }\left(s_{k}\right)=r_{k}^{\mu },\;k=1,2\\ u^{\mu }\left(s_{k}\right)=u_{k}^{\mu },\;k=1,2 \end{array}\right\}.$$

Notice that the generic variational functions $u^{\mu }\left(s\right)$ in ${\left\{r^{\mu },u^{\mu }\right\}}_{S}$ are not required to satisfy the kinematic constraint $u^{\mu }\left(s\right)u_{\mu }\left(s\right)=1$, while the line element ds is by construction required to be determined by Equation (117) in which $r^{\mu }\left(s\right)$ is an extremal curve (see definition below). Therefore, the synchronous variational principle realizes an unconstrained Lagrangian principle for classical mechanics. This principle is analogous to the unconstrained metric Lagrangian principle introduced above for EFE. Here, $r^{\mu }\left(s\right)$ and $u^{\mu }\left(s\right)$ are considered independent, so that $\delta r^{\mu }\left(s\right)$ and $\delta u^{\mu }\left(s\right)$ are independent too. In this case, it is immediate to show that the corresponding synchronous Hamilton variational principle takes the form

(125) $$\delta S_{p_{S}}\left(r,u\right)\equiv {\left.\frac{d}{d\alpha }\Psi \left(\alpha \right)\right|}_{\alpha =0}=0,$$

to hold for arbitrary independent displacements $\delta r^{\mu }\left(s\right)$ and $\delta u^{\mu }\left(s\right)$. Here,Ψ(α) is the smooth real function $\Psi \left(\alpha \right)=S_{p_{S}}\left(r+\alpha \delta r,u+\alpha \delta u\right)$, being $\alpha \in \left[-1,1\right]$ to be considered independent of r(s), $u\left(s\right)$ and *s*. In this case, the corresponding Euler–Lagrange equations deliver

(126) $$\begin{array}{ccc} \frac{\delta S_{pS}\left(r,u\right)}{\delta r^{\mu }\left(s\right)} & \equiv & \frac{D}{Ds}u_{\mu }-qF_{\mu \nu }u^{\nu }=0, \end{array}$$

(127) $$\begin{array}{ccc} \frac{\delta S_{pS}\left(r,u\right)}{\delta u^{\mu }\left(s\right)} & \equiv & u_{\mu }-g_{\mu \nu }\frac{dr^{\nu }\left(s\right)}{ds}=0, \end{array}$$

which can be combined to recover Equation (122) and imply also the kinematic constraint $u^{\mu }\left(s\right)u_{\mu }\left(s\right)=1$ to hold for extremal curves. Then, Equations (126) and (127) determine the extremal curves $r^{\mu }\left(s\right)$ and $u^{\mu }\left(s\right)$ which belong to the functional class ${\left\{r^{\mu },u^{\mu }\right\}}_{S}$ and are solutions of the same equations.

From this treatment, it follows that the meaning of unconstrained Lagrangian principles (or equivalently, synchronous principles) has a wider validity that is not restricted only to the variational theory for EFE. This proves the transversal relevance of the unconstrained approach, which arises as a fundamental connotation of the nature of variational theory in discrete and continuum classical field theory. It is important to underline in this respect the following remarks on the connection between synchronous principles in classical mechanics and gravitational field theory:

(1) A basic feature of the synchronous approach lies in the adoption of superabundant variables, which coincide only for extremal curves.

(2) The two approaches are similar for the treatment of the differential integration element, respectively, ds and dΩ, which are held fixed in the synchronous principles, in the sense that δds=0 and δdΩ=0.

(3) The normalization constraint is satisfied identically only by the extremal curves and not by generic variational (i.e., virtual) curves of ${\left\{r^{\mu },u^{\mu }\right\}}_{S}$. Therefore, Equation (125) should be regarded in a proper sense as an unconstrained variational principle.

(4) A crucial physical motivation behind the adoption of synchronous variational principles rather than asynchronous ones lies in the fact that only the unconstrained synchronous Lagrangian approach permit to achieve a corresponding Hamiltonian variational formulation (see discussion below).

### 8.2. Unconstrained Hamiltonian Principles

The second aspect of physical relevance that characterizes the unconstrained Lagrangian principles concerns the possibility of admitting corresponding unconstrained Hamiltonian theories. The issue is not a marginal one and has relevant potential implications both in classical and quantum gravity. In fact, first we notice that, given a Lagrangian principle, the existence of a Hamiltonian principle could not represent a compelling fact. Indeed, either in classical mechanics or continuum field theories, one must prove the existence of a Hamiltonian structure and a Hamiltonian theory starting from a Lagrangian formulation. In addition to this, it is well-known that unconstrained Lagrangian principles are always to be preferred with respect to constrained ones, since the treatment of constraints to fix the Hamiltonian structure of a theory might be a difficult task. Hence, unconstrained principles are better suited for the establishment of classical Lagrangian and Hamiltonian theories. More specifically, in the case of Lagrangian treatment of EFE the most famous historical approach to the problem was based on the constrained Hilbert–Einstein approach, and its fundamentals were due to the pioneering work by Dirac, who first developed the theory of constrained Hamiltonian dynamics. The latter violates PMC, and therefore it represents intrinsically a non-manifestly covariant Hamiltonian theory. A comprehensive presentation of the subject can be found for example in Ref. [17].

However, the proof of existence of a class of unconstrained Lagrangian variational principles yielding EFE provides a novel framework for the investigation about the formulation of unconstrained Hamiltonian theories underlying GR and the space-time metric tensor dynamics. Given the validity of PMC by the unconstrained Lagrangian framework, also the associated Hamiltonian theory should be characterized by the same manifest-covariance character. In turn, the subject can certainly have relevant implications in quantum gravity theory for the problem of mathematical and physical quantization of gravitational field and its connection with classical GR theory. The issue has been already recently proposed and discussed in the framework of synchronous Lagrangian variational principles realized by the unconstrained metric and Ricci theories (see Refs. [13,14] respectively). Nevertheless, given the proof of existence of an entire class of unconstrained principles, it is useful to recall the basic formalism for the establishment of corresponding Hamiltonian theories.

In the case of continuum fields, the appropriate formalism is provided by the DeDonder–Weyl Lagrangian and Hamiltonian treatments [11,12]. Such an approach was originally formulated for fields defined on the Minkowski space-time. The setting provided by unconstrained Lagrangian principles for EFE, together with the intrinsic validity of manifest covariance, permit the straightforward extension of the DeDonder–Weyl formalism to include also the gravitational field dynamics. In full generality, the starting point is the definition of the unconstrained variational Lagrangian function. Using a compact notation and without possibility of misunderstandings, the latter is denoted by $L\left(Z,\hat{\nabla }Z,\hat{Z}\right)\equiv L\left(Z_{\mu \nu },{\hat{\nabla }}_{\alpha }Z_{\mu \nu },{\hat{Z}}_{\mu \nu }\right)$ and is assumed for completeness to depend on the tensorial variational field $Z_{\mu \nu }$, its covariant derivative ${\hat{\nabla }}_{\alpha }Z_{\mu \nu }$ and a set of extremal tensor fields ${\hat{Z}}_{\mu \nu }$. The corresponding canonical momenta are defined as in classical mechanics as $\Pi \equiv \Pi_{\mu \nu }^{\alpha }=\frac{\partial L\left(Z,\hat{\nabla }Z,\hat{Z}\right)}{\partial \left({\hat{\nabla }}_{\alpha }Z^{\mu \nu }\right)}$ so that the canonical state can be represented as $\left\{x\right\}=\left\{Z,\Pi \right\}$. The Hamiltonian density $H=H\left(x,\hat{x}\right)$ associated with the Lagrangian $L\left(Z,\hat{\nabla }Z,\hat{Z}\right)$ is then provided by the Legendre transform

(128) $$L\left(Z,\hat{\nabla }Z,\hat{Z}\right)\equiv \Pi_{\mu \nu }^{\alpha }{\hat{\nabla }}_{\alpha }Z^{\mu \nu }-H\left(x,\hat{x}\right).$$

Then, given the definition of a suitable functional class of variations for the canonical state variables, the Hamiltonian action functional is written as

(129) $$\begin{array}{ccc} S_{H}\left(x,\hat{x}\right) & = & \int d\Omega L\left(x,\hat{x}\right)\\ & = & \int d\Omega \left[\Pi_{\mu \nu }^{\alpha }{\hat{\nabla }}_{\alpha }Z^{\mu \nu }-H\left(x,\hat{x}\right)\right], \end{array}$$

while the corresponding synchronous Hamiltonian variational principle becomes

(130) $$\delta S_{H}\left(x,\hat{x}\right)\equiv {\left.\frac{d}{d\alpha }\Psi \left(\alpha \right)\right|}_{\alpha =0}=0,$$

which is defined in terms of the Frechet derivative and is required to hold for arbitrary independent variations $\delta Z^{\mu \nu }$ and $\delta \Pi_{\mu \nu }^{\alpha }$ in the respective functional classes. The corresponding variational derivatives yield the continuum Hamilton equations

(131) $$\begin{array}{ccc} \frac{\delta S_{H}\left(x,\hat{x}\right)}{\delta Z^{\mu \nu }} & \equiv & -\frac{\partial H\left(x,\hat{x}\right)}{\partial Z^{\mu \nu }}-{\hat{\nabla }}_{\alpha }\Pi_{\mu \nu }^{\alpha }=0, \end{array}$$

(132) $$\begin{array}{ccc} \frac{\delta S_{H}\left(x,\hat{x}\right)}{\delta \Pi_{\mu \nu }^{\alpha }} & \equiv & {\hat{\nabla }}_{\alpha }Z^{\mu \nu }-\frac{\partial H\left(x,\hat{x}\right)}{\partial \Pi_{\mu \nu }^{\alpha }}=0. \end{array}$$

Written explicitly, these become

(133) $$\begin{array}{ccc} {\hat{\nabla }}_{\alpha }\Pi_{\mu \nu }^{\alpha } & = & -\frac{\partial H\left(x,\hat{x}\right)}{\partial Z^{\mu \nu }}, \end{array}$$

(134) $$\begin{array}{ccc} {\hat{\nabla }}_{\alpha }Z^{\mu \nu } & = & \frac{\partial H\left(x,\hat{x}\right)}{\partial \Pi_{\mu \nu }^{\alpha }}, \end{array}$$

which are equivalent to the Euler–Lagrange equation provided by the Lagrangian principle.

This short derivation shows the convenience of implementing unconstrained Lagrangian principles as far as the construction of corresponding Hamiltonian theories is concerned. The DeDonder–Weyl formalism is simultaneously consistent with classical mechanics and PMC, while the absence of constraints makes the theory easy to implement and understandable from the physical point of view. In conclusion, this feature represents a further advantage of the implementation of unconstrained principles in place of constrained and non-manifestly covariant ones. Remarkably, this feature pertains both classical and quantum gravity theories, see for example the discussion proposed in Ref. [3].

## 9. Conclusions

A fundamental requisite that should be satisfied by physical laws of classical, quantum and relativistic mechanics as well as continuum field theories concerns the possibility of expressing the dynamical equations in terms of least-action variational principles. In fact, the existence of variational formulations is usually considered a property of mathematical consistency and correctness for the same physical laws. In particular, the representations in terms of Lagrangian and Hamiltonian settings are essential to understand the physical properties of classical and quantum fields, including, for example, their degrees of freedom and gauge properties, the role of constraints, the disclosure of symmetries and conservation laws.

In this paper, a systematic theoretical formulation of Lagrangian variational principles yielding the continuum gravitational field dynamics of classical General Relativity (GR) has been presented. The problem has been cast in the framework of validity of the Principle of Manifest Covariance (PMC), namely the requirement that variational and extremal (i.e., observable) fields and equations must exhibit a 4-tensor character with respect to the group of local point transformations characteristic of GR theory. A general formalism for the construction of Lagrangian functions and corresponding action principles yielding the Einstein field equations (EFE) has been illustrated. As a remarkable aspect, it has been shown that the latter equations can be equivalently obtained through multiple Lagrangian functions exhibiting different physical meanings and mathematical connotations. The corresponding variational principles can be classified in two categories, respectively, referred to as constrained and unconstrained Lagrangian principles. They are distinguished on the basis of the constraints that can possibly be imposed on variational tensor fields, to be realized, for example, by normalization or orthogonality conditions.

Explicit realizations of several Lagrangian principles have been proposed, which are referred to here as Ricci, metric-tensor and tangent 4-vectors Lagrangian principles. For all of them, it has been proved that only the unconstrained framework can satisfy PMC correctly and reproduce EFE as extremal equations. Remarkably, the synchronous variational principle recently disclosed in Ref. [23] has been shown to belong to the unconstrained category. As discussed here, due to its physical meaning and the formal analogy with the case of classical mechanics, this also provides a promising route for the formulation of a consistent Hamiltonian theory and corresponding canonical quantum description of the gravitational field. In contrast, it has been shown that the original Hilbert–Einstein formulation can only be couched in the framework of a constrained Lagrangian principle together with the condition of violation of PMC. As a consequence, the Hilbert–Einstein theory arises as a constrained principle that is intrinsically non-manifestly covariant, with critical implications for the formulation of a corresponding constrained Hamiltonian theory based on the Dirac theory of constrained dynamics.

The outcome of the present research permits us to state the excellence of the unconstrained Lagrangian principles as preferred settings for the variational formulation of EFE, with respect to the constrained ones. The conclusion is in agreement with the tensorial mathematical structure and conceptual meaning of GR. The unconstrained variational setting therefore arises as a natural framework for the Lagrangian formulation of classical EFE, with notable implications on the corresponding Hamiltonian theory and the consequent establishment of a consistent canonical quantum gravity theory.

## Data Availability

All data pertinent to this study are contained in the paper.
